# Snake envenomation in veterinary medicine: comparative insights and emerging therapies

**DOI:** 10.3389/fvets.2026.1750963

**Published:** 2026-01-21

**Authors:** Alessandro Migliorisi, Tyler Johnson, Tatum Nelson, George L. Elane, Yu Ueda, Kallie J. Hobbs

**Affiliations:** 1 Roaring Fork Equine Medical Center, Glenwood Springs, CO, United States; 2 College of Veterinary Medicine, North Carolina State University, Raleigh, NC, United States; 3 College of Veterinary Medicine and Biomedical Sciences, Texas A&M University, College Station, TX, United States

**Keywords:** antivenom, envenomation, human, snake, venom

## Abstract

Snakebite envenomation poses a significant threat to both public health and animal welfare, resulting in substantial human suffering and economic burden worldwide. Recognized by the World Health Organization as a neglected tropical disease, snakebites disproportionately affect impoverished rural regions across Africa, Asia, and South America, with an estimated 2.7 million envenomations and 81,000–138,000 deaths annually. In veterinary medicine, snakebites are similarly impactful, with up to 300,000 animals affected each year in the United States alone—primarily dogs and cats—while global veterinary cases likely number in the millions. Despite this, snakebites remain non-notifiable diseases, contributing to significant underreporting. The economic implications are profound, with treatment costs for human victims exceeding $200,000 per case and veterinary care ranging from $8,000 to $50,000 per case, often surpassing the financial capacity of pet owners. Beyond acute care, long-term sequelae such as chronic neuropathy and tissue damage further compound the burden. Current literature is limited in comparative analyses of envenomation mechanisms across species, particularly in livestock. This review will create a deeper understanding of pathophysiology, treatment modalities, and emerging therapies. Understanding of this background is essential to further advancements in science surrounding snake envenomation in both human and veterinary species.

## Introduction

Snakebites pose a significant threat to public health and animal welfare inflicting substantial human and economic costs. According to the World Health Organization snakebite envenoming is a neglected tropical disease that is responsible for enormous suffering, disability and premature death around the globe and particularly in impoverished rural areas in Africa, Asia and South America As a result, adding up to about 2.7 million cases of envenoming and 81,000–138,000 deaths a year (1). Each year, between 150,000 and 300,000 animals are bitten by venomous snakes in the United States alone, with dogs and cats accounting for an estimated 150,000 cases. Because snakebites are not notifiable diseases, there is likely a significant underestimation of envenomation cases each year, and the worldwide number of veterinary snakebite cases likely reaches into the millions annually, especially in regions with high snakebite incidence such as South America, Africa and Asia. While precise veterinary data remains limited, snake envenomation is recognized as a leading cause of intensive care unit admissions among companion animals, as well as of loss of livestock in rural areas and of economic impact, further highlighting the pervasive impact of this issue. Currently, most studies focus on dogs, cats and horses, and only sparse information is available on the impact that snakebites have on livestock species (2).

The financial burden associated with snakebite treatment can be substantial and often insurmountable. For human victims, the average treatment cost can exceed $200,000 per case, placing a significant strain on individuals and families (3, 4). In companion animals veterinary medicine, the economic impact is equally concerning, with treatment costs ranging from $8,000 to $50,000, often exceeding the financial capacity of pet owners (Texas A&M and North Carolina State University medical records). Cost of intensive care, multiple antivenom vials used, and mechanical ventilation all contribute to significant expenses in envenomated dogs and cats. This disparity in treatment accessibility underscores a critical need for more affordable and effective therapeutic options for both human and animal patients.

Two of the most affected veterinary species in the literature are dogs and horses, this is most likely due to their inquisitive nature and sharing of habitat with areas that are common for snakes (5). In North America the primary culprits behind the alarming statistics for snakebites are a select group of venomous snakes belonging to two families, Viperidae and Elapidae. While human victims often have access to insurance coverage, which can mitigate the financial burden of treatment, veterinary patients frequently lack such support, leaving owners facing devastating medical bills and difficult decisions regarding their pet’s care.

Beyond the immediate financial consequences, snakebite envenomation can have severe and long-lasting health implications. Even with the most advanced medical care available, human patients often experience debilitating sequelae, including chronic neuropathy and significant tissue damage. These debilitating conditions can significantly impact an individual’s quality of life and overall wellbeing.

Currently there is a paucity in the literature on the comparative mechanisms of envenomation across both human and animal species, therefore, the goal of this review is also to discuss the effects that different classes of venom toxins can have using a comparative approach and clinical elements. A deeper understanding of pathophysiology, current treatment options, and novel therapies will help to bridge this gap for future research.

## Pathophysiology of snake envenomation

**Figure 1:**
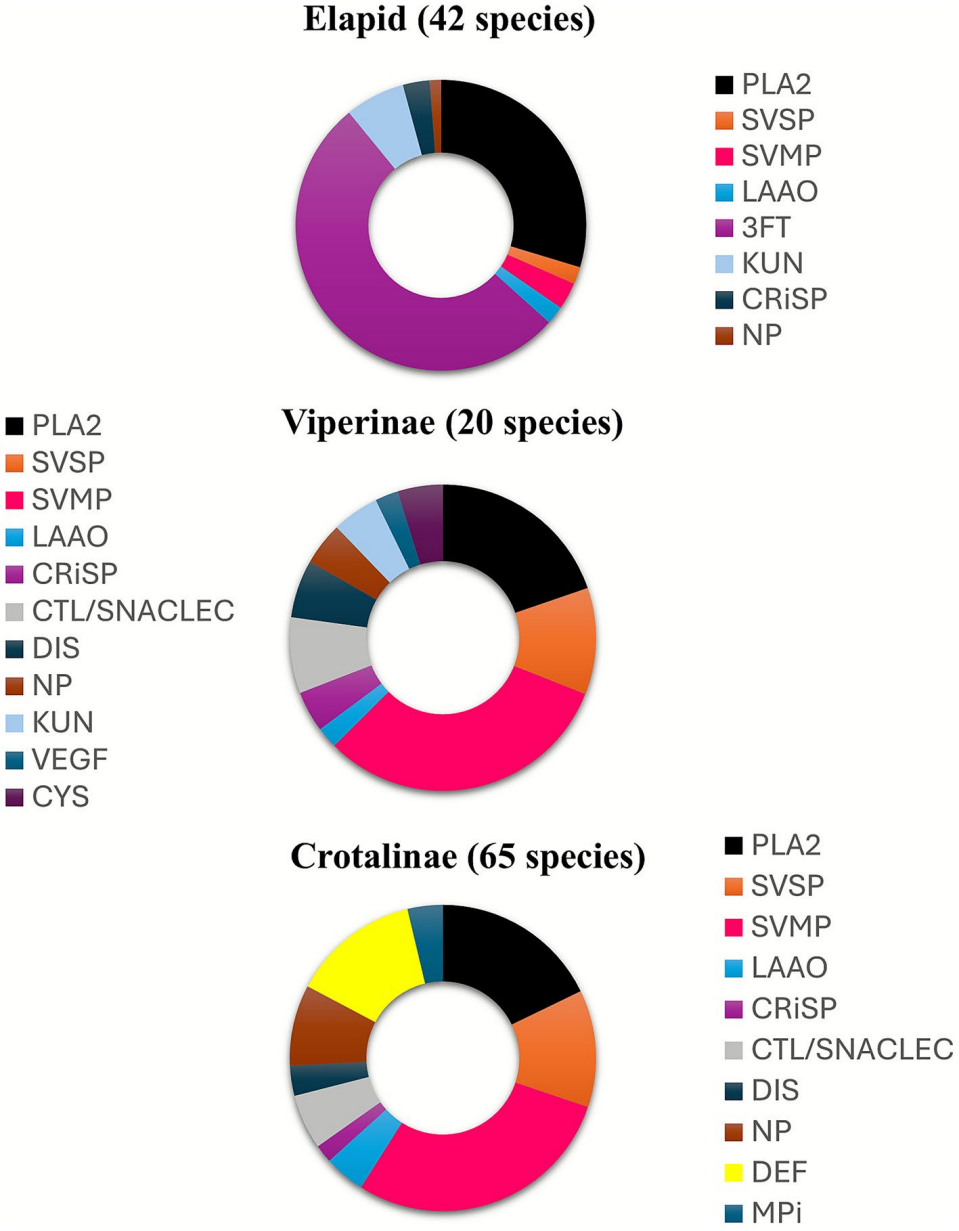
The relative proportions of different protein families for the venoms of elapids, viperines, and crotalines, averaged from the number of species noted in the brackets. 3FTx, three-finger toxin; CRiSP, cysteine-rich secretory protein; CTL, C-type lectin; CYS, cystatin; DEF, defensin (crotamine); DIS, disintegrin; KUN, kunitz peptide; LAAO, L-amino acid oxidase; MPi, snake venom metalloprotease inhibitor; NGF, nerve growth factor; NP, natriuretic peptide; PLA_2_, phospholipase A_2_; SVMP, snake venom metalloprotease; SVSP, snake venom serine protease; VEGF, vascular endothelial growth factor.

The pathophysiology of snake envenomation is complex and multifaceted. Snake venom is an elaborate mixture of enzymatic and non-enzymatic peptides designed to immobilize prey and begin the digestive process before the snake consumes it. There are different types of venom toxins including cytotoxic, neurotoxic, myotoxic, and hemotoxic varieties, and each type affects the body in distinct ways (Table 1). The severity of envenomation in each patient depends on elements related to both the snake and the victim. The final characteristics of the venom composition are influenced by a variety of factors including the snake family, genus, species, geographical location, time of the year, type of prey available, snake’s age, and time from the last meal (6). The volume of venom injected is also an important determining factor. The location of the bite, as well as the size and health status of the envenomated patient, are also likely to influence the severity of the clinical picture. The complexity and variability in venom composition among, and within, snake species may create some degree of confusion. Figure 1 illustrates the relative proportions of toxin families in three medically important snake families and sub-families (7). As the pathophysiology of snake envenomation is well described in the literature this section will only cover the major highlights of the most prominent damaging factors in snake envenomation.

**Table 1:** Main families of snake venom toxin.

Venom toxin class	Mechanism(s) of action	Possible clinical effects
Cytotoxins (3FTx)	Non-enzymatic—cell membrane dysfunction	Tissue necrosis, inflammation, local and systemic hemorrhage, coagulopathy, hemolysis, cardiac and skeletal muscle damage, neuromuscular blockade (muscle paresis, paralysis, hypoventilation)
Metalloproteinases	Enzymatic—Extracellular matrix and capillary basement membrane degradation; Fibrinogenolytic; Platelet function inhibition; Endotheliocytes apoptosis	Local tissue swelling, inflammation, local and systemic hemorrhage, coagulopathy
Disintegrins (structurally related to metalloproteinases)	Non-enzymatic—Blocking of cell–cell and cell-matrix interactions	Platelet aggregation inhibition often leading to local and systemic hemorrhage
Hyaluronidase	Enzymatic—Hydrolysis of hyaluronic acid	Local tissue swelling, inflammation
Phospholipases A2	Enzymatic—Hydrolysis of cell membrane phospholipids	Clinical effects depend on affinity for specific tissue receptors. Local and systemic myotoxicity, cardiotoxicity, neuromuscular blockade (muscle paresis, paralysis, hypoventilation), hemolysis, coagulopathy are possible
Serine proteases	Enzymatic—Numerous and variable mechanisms of action reported resulting in pro or anticoagulant activity	Local and systemic hemorrhage, hypotension
Cysteine-rich secretory proteins (CRiSP)	Non-enzymatic—Ion channel blocking; Increased vascular permeability; Proinflammatory	Local tissue swelling
L-amino acid oxidase (LAAO)	Enzymatic—Generation of H_2_O_2_ and oxidative stress damage	Tissue necrosis, inflammation
Kunitz peptides (KPs)	Non-enzymatic—Neurotoxic KPs: ion channel blocking, modulation of neuromuscular transmission (stimulatory or inhibitory effect). Non-neurotoxic KPs: inhibition of serine proteases involved in coagulation	Neurotoxicity, coagulopathy, inflammation

## Cytotoxins and neurotoxins

**Figure 2:**
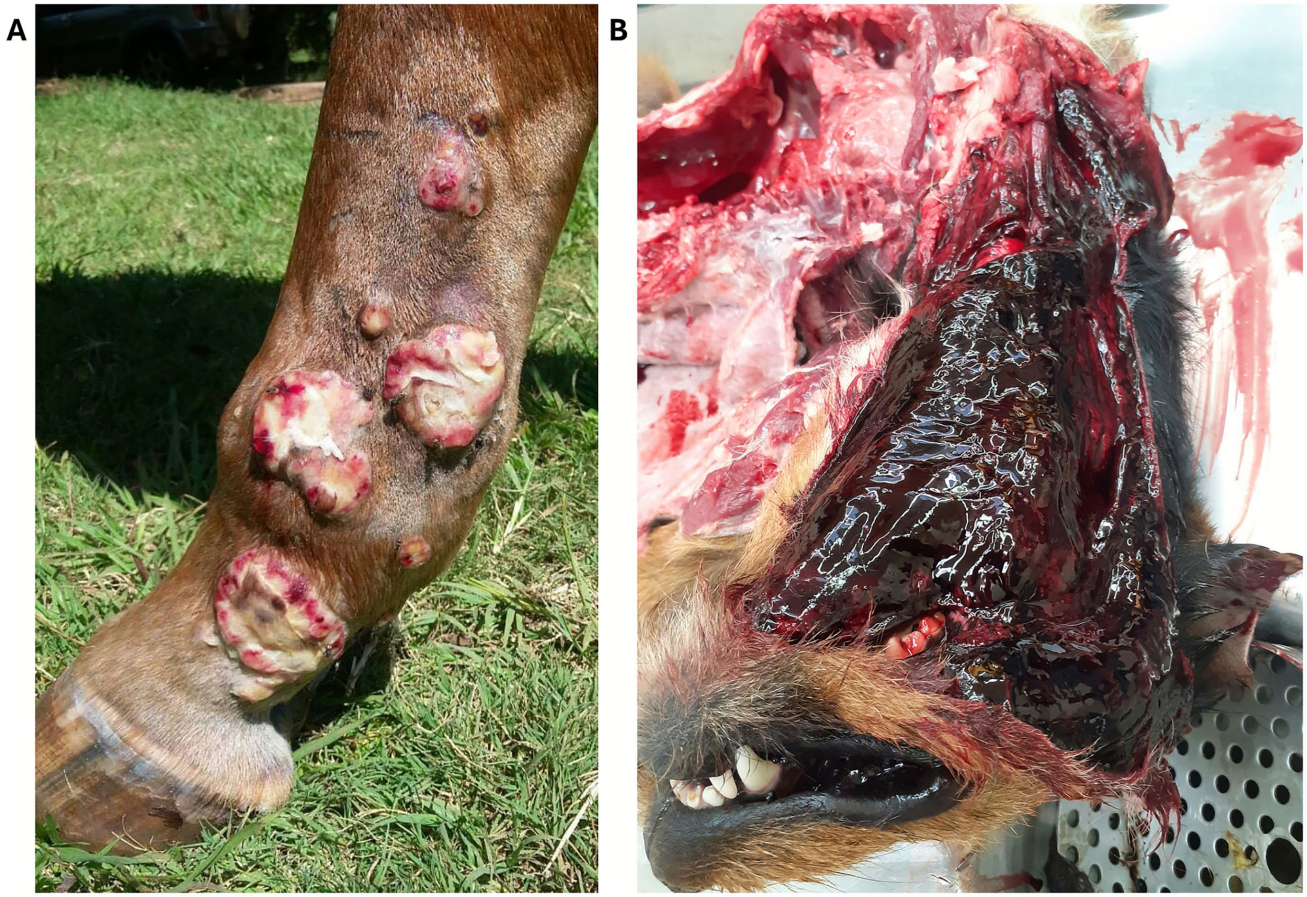
**(A)** Snakebite site on a horse envenomated by a Mozambique spitting cobra (*Naja mossambica*) in Malawi. The “skipping” fashion of lesions, leaving areas of normal skin between necrotized skin, is also typically observed in human patients. Courtesy of Dr. Bird, BVSc. **(B)** Postmortem evidence of diffuse tissue hemorrhage over head and neck regions in a dog envenomated on its head by a puff adder (*Bitis arietans*) in South Africa. Courtesy of Dr. Williams, BVSc, Pathology Department, University of Pretoria, South Africa.

**Figure 3:**
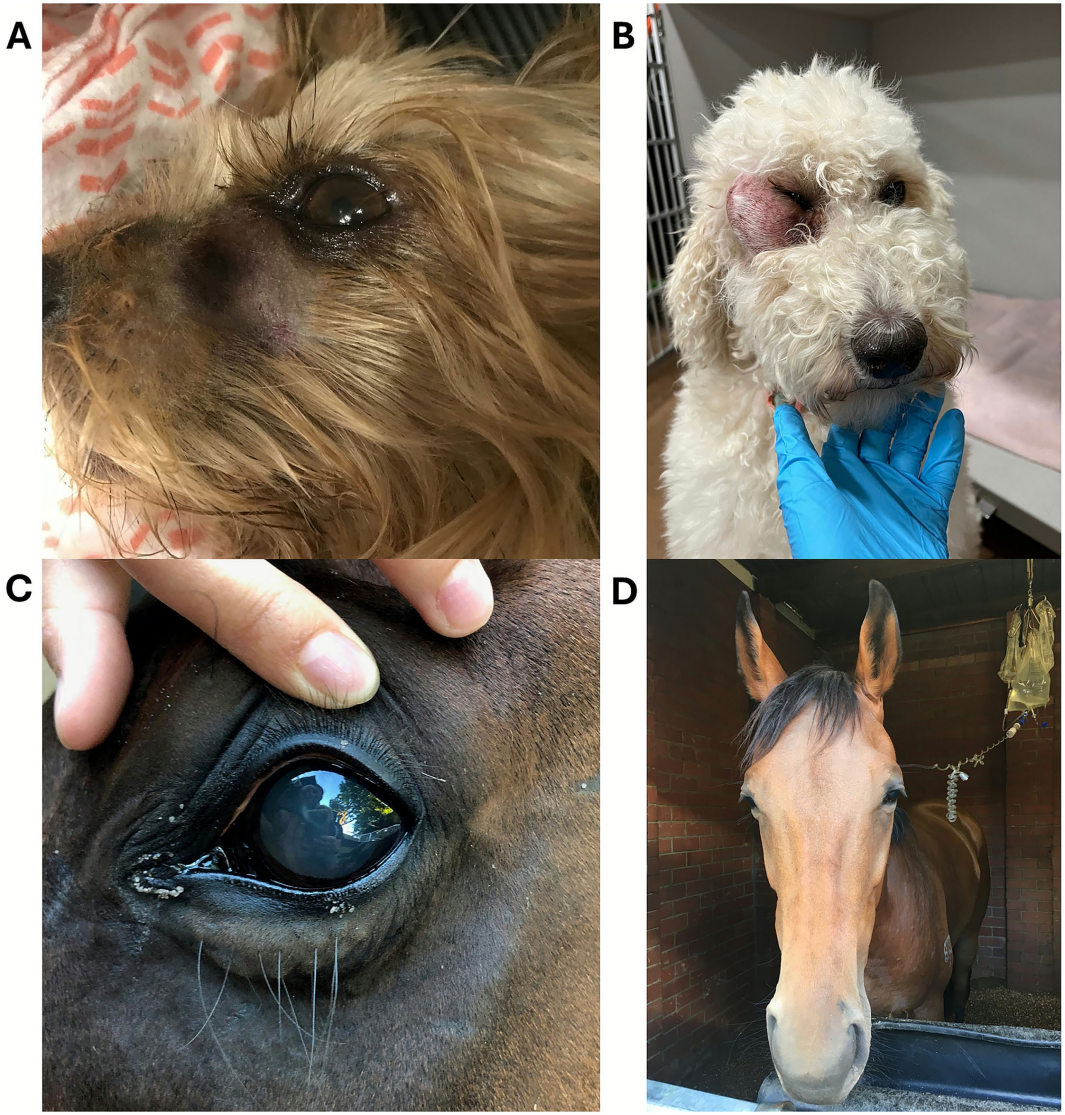
**(A)** Periocular Viperidae snakebite in a dog in Colorado, USA. Courtesy of Dr. Rutten, DVM, DACVECC, Colorado State University, USA. **(B)** Miosis in a dog envenomated by a Viperidae snake in Colorado. Courtesy of Dr. Corsi, DVM, DACVECC, Wheat Ridge Animal Hospital, USA. Mydriasis **(C)** and bilateral palpebral ptosis **(D)** in horses envenomated by tiger snakes (*Notechis scutatus*) in Australia. Courtesy of Dr. Cullimore, MVB, MANZCVS, DACVIM, Ascot Equine Veterinarians, Australia.

Cytotoxins are a highly diverse group of toxins, primarily non-enzymatic toxins that are structurally characterized by the presence of loops arranged in the shape of a three-fingered fold (8). Cytotoxins are commonly found in venomous snakes, including rattlesnakes, Russell’s vipers, and puff adders, and are major contributors to snakebite-related morbidity and long-term disability. Many cytotoxins cause widespread cellular damage due to the high binding affinity for membrane phospholipids and their pore-forming and membrane-lytic action. This results in damage of epithelial cells, skeletal and cardiac myocytes, and erythrocytes, therefore leading to tissue necrosis, local hemorrhage, hemolysis and overall severe physiological dysfunction (9, 10). Because lytic cytotoxins act directly on cells and tissues, they disrupt their structural integrity and trigger inflammatory and degenerative processes. The Mozambique spitting cobra (*Naja mossambica*) exemplifies cytotoxic venom, often causing superficial necrosis in humans and horses (Figure 2A) (11). In dogs, periocular bites can result in corneal ulceration, keratomalacia, or enucleation (Figure 3B) (12). Some cytotoxins also modulate membrane-bound enzymes, depolarize cardiomyocytes and neurons, and inhibit platelet aggregation (13–15). Additionally, L-amino acid oxidase (LAAO) enhances cytotoxicity via oxidative stress (16).

**Figure 4:**
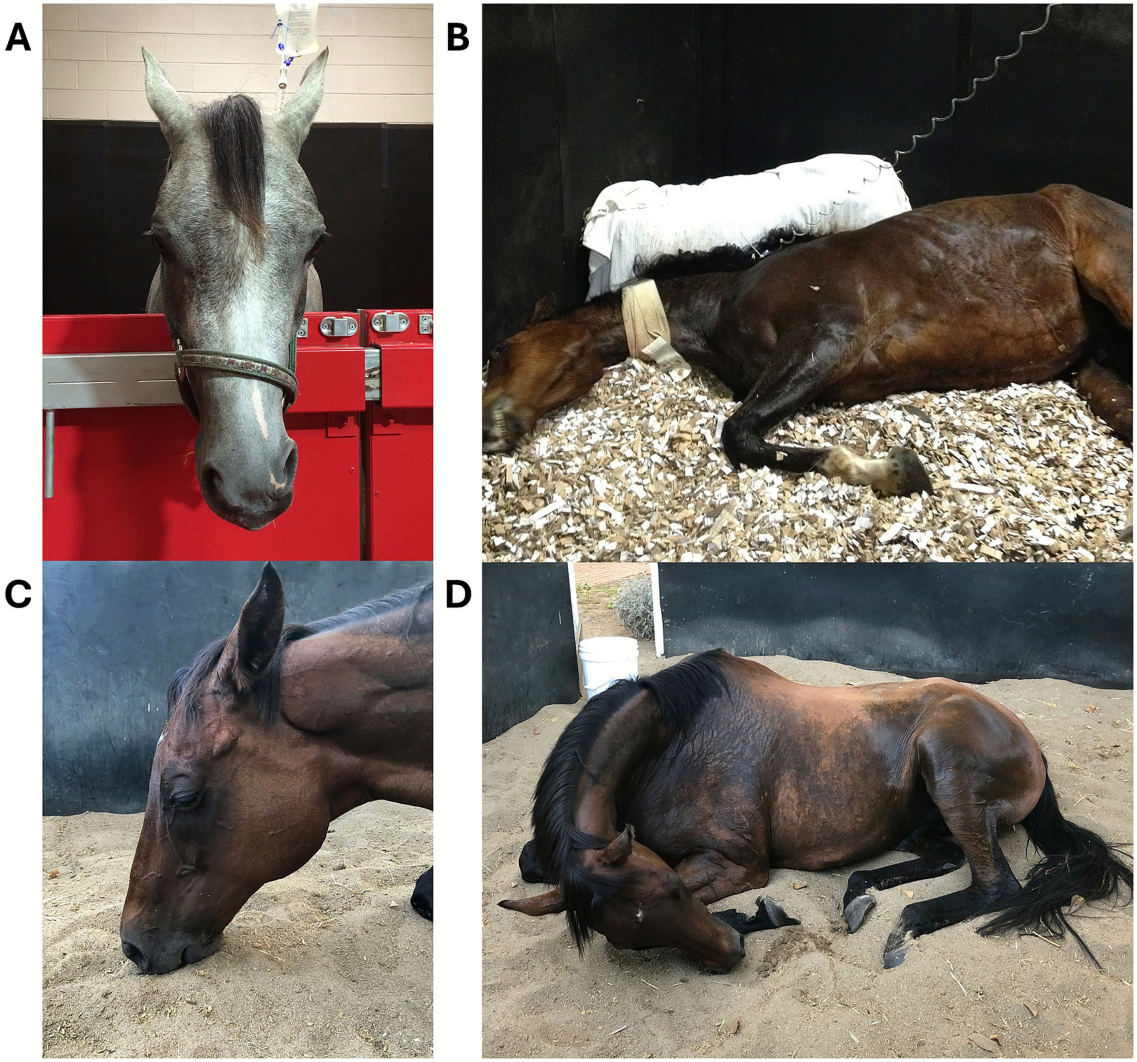
Elapid envenomation in Australia. **(A)** Confirmed tiger snake (*Notechis scutatus*) envenomation in an Arabian filly, with bite on the right side of the muzzle resulting in unilateral facial nerve paralysis without discernible swelling. **(B)** Recumbency in a presumptive eastern brown snake (*Pseudonaja textilis*) envenomation. The horse regained normal standing function within an hour of receiving antivenom. Courtesy of Dr. Bamford, BVSc, DACVIM, The University of Melbourne, Australia. Obtundation **(C)** and recumbency **(D)** in horses envenomated by tiger snake (*Notechis scutatus*) in Australia. Courtesy of Dr. Cullimore, MVB, MANZCVS, DACVIM, Ascot Equine Veterinarians, Australia.

Many cytotoxins also exhibit neurotoxic activity as three-finger toxins (3FTx), which impair neuromuscular transmission. Alpha-neurotoxins block nicotinic acetylcholine receptors, while fasciculins inhibit acetylcholinesterase, prolonging muscle stimulation (17, 18). Elapid snakes (e.g., cobras, coral snakes, taipans) typically produce neurotoxic venoms that act rapidly, though onset in dogs can be delayed up to 18 h (19). Clinical signs include lethargy, vomiting, ptyalism, generalized weakness, paralysis, and hypoventilation; ocular signs such as ptosis and transient megaesophagus have also been reported. Coral snake envenomation may cause hemolysis and renal injury. In horses, Elapid bites—more common in Africa and Australia—present as neurotoxicity without local swelling (17–22) (Figure 4).

Certain Viperid species, such as Mojave and Timber rattlesnakes, produce beta-neurotoxic PLA₂s (e.g., crotoxin, taipoxin) that inhibit acetylcholine release, causing irreversible paralysis. Neurotoxic syndromes vary, with alpha and beta toxins inducing flaccid paralysis, etc. Ophthalmic abnormalities, including ophthalmoplegia and ptosis, occur in multiple species (Figures 3A–D). Although neurotoxic venoms are highly lethal, their components have inspired drug development for pain and other conditions; for instance, mambalgins and α-cobratoxin exhibit analgesic properties, with α-cobratoxin approved for pain management in China (23, 24).

## Metalloproteinases

Snake venom metalloproteinases (SVMPs) and hyaluronidases, often termed “spreading factors,” are predominantly found in Viperid snakes, especially the Crotalinae subfamily (25), with lower concentrations in Elapid and Colubrid venoms. These enzymes disrupt tissue architecture and reduce interstitial viscosity, facilitating toxin diffusion. SVMPs exert hemorrhagic, fibrinolytic, and proapoptotic effects and are classified by size into three groups: P-I (20–30 kDa), P-II (30–60 kDa), and P-III (60–100 kDa) (26). P-I SVMPs degrade extracellular matrix components such as collagen and laminin, causing capillary damage, edema, blistering, and local hemorrhage that may progress systemically. Examples include Ht-D from *Crotalus atrox*, H2-proteinase from *Trimeresurus flavoviridis*, and fibrolase from *Agkistrodon contortrix*. P-II SVMPs, found only in Viperids, exhibit hemorrhagic and fibrinolytic activity and inhibit platelet function; notable toxins include bilitoxin-1 (*Agkistrodon bilineatus*) and ahpfibrase (*Gloydius halys*) (27–29). Many P-II SVMPs possess disintegrin domains that block platelet aggregation. P-III SVMPs, present in Viperid, Elapid, and Colubrid venoms, degrade extracellular matrix, disrupt hemostasis, and induce endothelial apoptosis, sometimes activating factors X and II. Disintegrins derived from SVMPs further impair platelet adhesion by binding αIIbβ3 receptors and inhibiting von Willebrand factor-mediated aggregation. These properties have inspired antiplatelet drugs such as eptifibatide and tirofiban, derived from the Dusky Pygmy Rattlesnake (*Sistrurus miliarius barbourin*) and the Saw Scaled Viper (*Echis carinatus*), respectively (30).

## Phospholipases A_2_

Venom PLA_2_ are enzymatic toxins found in the venom of Colubrid, Elapid and Viperid snakes. The PLA₂s hydrolyze phospholipids in cell membranes, causing membrane destabilization and cell lysis, and promoting the release of pro-inflammatory mediators. Snake PLA_2_s display a wide range of biological activities including pre and post synaptic neurotoxicity, local and systemic myotoxicity, cardiotoxicity, hemolytic and coagulopathic effects, and the overall ability to induce organ (lungs, liver, kidney, testis, pituitary) damage (31). Each PLA_2_ exhibits affinity for a particular tissue and, within tissues, for specific target proteins or glycoproteins. The presence of these target sites explains the specific biological effects of each PLA_2_, as well as the different prey susceptibility to snake venoms (32). Several target proteins have been identified, such as the voltage-sensitive K^+^ channel for β-bungarotoxin (33), the neuronal pentraxin and taipoxin-associated Ca^2+^binding protein (TCBP-49) for taipoxin (34), an M-type receptor from skeletal muscles for OS_1_ and OS_2_ (two PLA_2_ found in the common Taipan [*Oxyuranus scutellatus*]) (35) and factor X for several anticoagulant PLA_2_ (36). The clinical consequences that originate from PLA_2_ are largely based on the type and the venom concentration of PLA_2_. Neurotoxicity, rhabdomyolysis, coagulopathies and hemolysis can all occur independently from one another.

## Serine proteases

The venom of Viperid and, in part, Elapid and Colubrid snakes is a rich source of serine proteases. These venom toxins mostly affect the coagulation cascade, the kallikrein-kinin system and platelet functions, therefore impairing the victim’s hemostasis (37). Snake venom serine proteases (SVSP) are enzymatic toxins that can have pro or anticoagulant properties. The final effects mostly depend on the type of injected toxin and the available substrates in the envenomated animal. SVSPs can exhibit thrombin-like activity, therefore promoting the conversion of fibrinogen into fibrin, which can induce platelet aggregation, or activate factors X, V, II, protein C and plasminogen (38–44). The ability of some SVSPs to activate the kallikrein-kinin system results in the release of bradykinin, a potent vasodilator, and the development of hypotension. Several Viperid snakes are known to cause hypotension through this type of SVSP (44–47). Another mechanism for hypotension in the envenomated victim is mediated by a different class of non-enzymatic venom toxins, mostly present in Elapid and Viperid snakes, and referred to as natriuretic peptides (NPs) (48). These NPs induce marked vasodilation and myocardial depression, eventually resulting in loss of consciousness (48).

## Clinical pathology of snake envenomation

Once injected, the various venom toxins will affect multiple body systems causing organ damage of variable degree. The pathological consequences on body systems can be detected through routine complete blood count (CBC), plasma biochemistry, as well as through quantification of circulating cardiac troponin and urine analysis. The extravasation of fluid in inflamed tissues explains the development of hemoconcentration often seen in envenomated dogs (49, 50). The hemoconcentration can also be enhanced by concurrent vomiting, diarrhea and splenic contraction. The incidence of hemolysis in veterinary patients seems to be overall infrequent. When hemolysis is present, the evidence on cytology of spherocytes helps support the action of venom PLA_2_ on erythrocytes and differentiates it from fragmentation hemolysis, which is more typical of disseminated intravascular coagulation (DIC) (51). Nevertheless, venom-induced hemolytic anemia can also manifest without spherocytes but with the more classic schistocytes (red cell fragments). Importantly, the presence of hemolysis, red blood cell fragments and thrombocytopenia, increases the risk for the development of snakebite-associated thrombotic microangiopathy, of which the more severe complication is acute kidney injury (52–54). Intravascular hemolysis is often reflected by the red discoloration of urine, although this could also indicate myoglobinuria (22, 55, 56). Transient echinocytosis on a peripheral blood smear can be found in envenomated dogs and cats, and a positive relationship between venom dose and the number of echinocytes has been shown *in vitro* (57–60). Erythroid loops are a transient finding of unknown significance observed on peripheral blood smears in envenomated dogs (61). Alterations in leukocyte counts are common following envenomation, and mostly reflect the patient’s stress response, characterized by neutrophilic leukocytosis, lymphopenia or both. Biochemical abnormalities are frequent and generally reflect the degree of organ damage. Increases in alanine amino-transferase (ALT), aspartate aminotransferase (AST), lactate dehydrogenase (LDH), and glutamate dehydrogenase (GLDH) support hepatocellular damage (50, 62, 63). Similarly, elevated concentrations of creatine kinase (CK), AST, and LDH indicate skeletal or cardiac muscle injury. Measurement of cardiac troponin is a reliable way to identify cardiac muscle injury in envenomated animals (50, 64–67). Envenomated patients with significant muscle injury or hemolysis are at higher risk of pigment (myoglobin and hemoglobin) nephropathy, and the development of acute kidney injury (AKI) is associated with increased mortality in envenomated dogs (68). Furthermore, direct renal epithelium cytotoxicity was demonstrated *in vitro* for the venom of some species of coral snakes, suggesting that AKI could develop independently of concurrent pigment nephropathy (69). Close monitoring of renal function in these patients should be implemented for an early identification of azotemia, proteinuria or urinary casts (70–73). In some instances, markers of renal function may remain within normal limits. For example, dogs envenomated by the European Adder (*Vipera berus*) did not show elevations in serum creatinine, while urinary markers of tubular injury, such as cystatin B, increased instead (74).

## Venom induced coagulopathy

Nearly all venomous snakes possess some type of toxin that alters the victim’s coagulation function, which can manifest as local (bite site) hemorrhage, localized tissue hemorrhage, or systemic coagulopathy. The interferences on hemostasis can lead to Venom-Induced Consumption Coagulopathy (VICC), a severe disorder characterized by the rapid consumption of clotting factors and platelets. Unlike DIC, VICC is typically not associated with widespread microthrombi formation but still results in a profound inability to clot. The inability to form a stable clot can also originate from the fibrinogenolytic activity of some venom toxins. In these instances, the resulting afibrinogenemia, for example seen after Western diamondback rattlesnake envenomation, does not indicate a primary consumptive coagulopathy (75). Consumption of coagulation factors and platelets contributes to the development of bleeding at the bite site, spontaneous bleeding, including mucosal hemorrhage and internal bleeding, and late-onset coagulopathy. VICC is a hallmark of envenomation by certain Viperid snakes and contributes significantly to morbidity and mortality if not promptly treated. In addition to SVMPs, PLA_2_ and SVSPs with coagulopathic effects, additional venom components that have a main influence on coagulation include the C-type lectin-like proteins (CLPs), also known as snacles, which can either promote or inhibit coagulation. Some CLPs bind to and inhibit factors IX, X and II, thereby exhibiting anticoagulant activity (76–78). Whereas others promote platelet aggregation (79–81). Given the numerous toxins that can affect the coagulation system with different mechanisms, the type of coagulopathy that develops following an envenomation can be quite variable and is mostly influenced by the snake species and its venom proteomic characteristics. Importantly, envenomated animals can have normal platelet counts but dysfunctional platelets.

**Figure 5:**
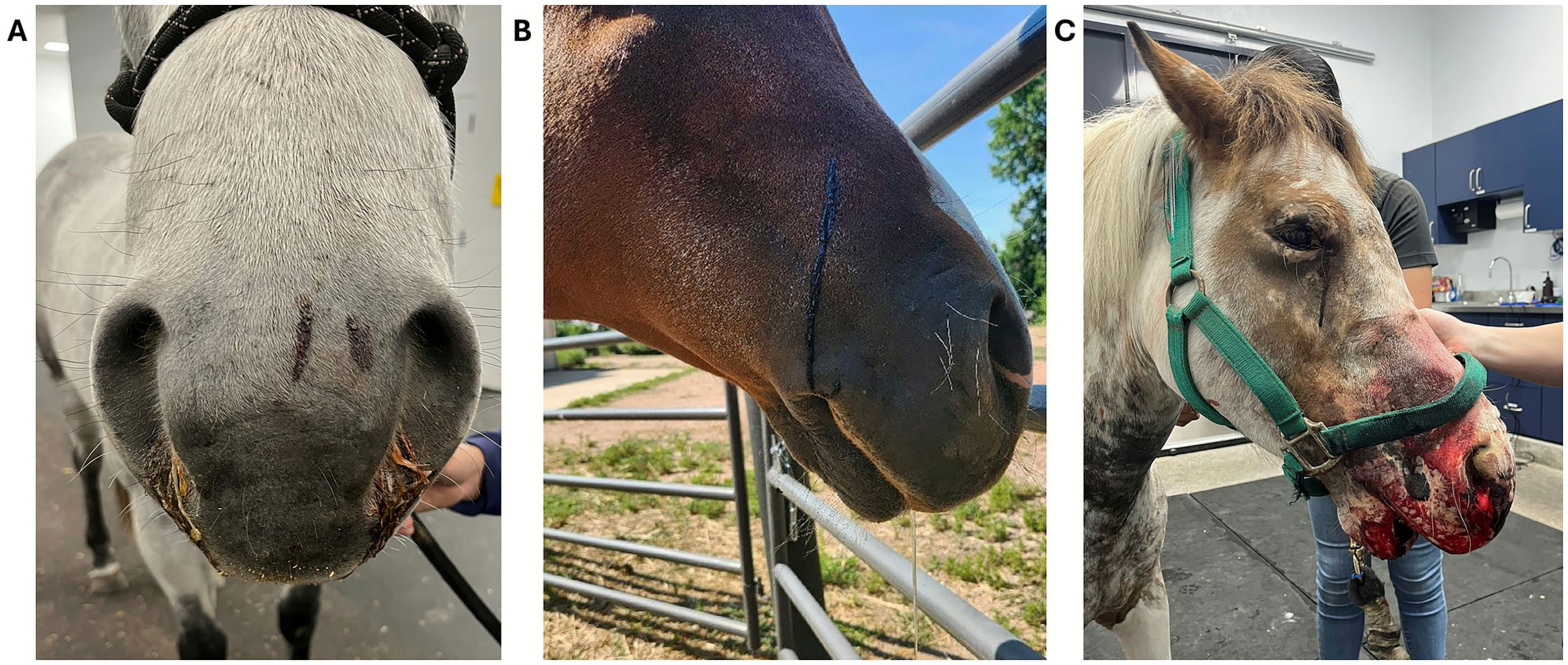
**(A,B)** Evidence of bleeding at the bite site in two horses envenomated in Colorado by prairie rattlesnakes (*Crotalus viridis viridis*). Courtesy of Dr. Migliorisi, DVM, DACVIM (LAIM), DACVECC, Roaring Fork Equine Medical Center, USA. **(C)** Epistaxis in a horse envenomated in Florida. Courtesy of Dr. Henderson, DVM, Peterson Smith Equine Hospital, USA.

**Figure 6:**
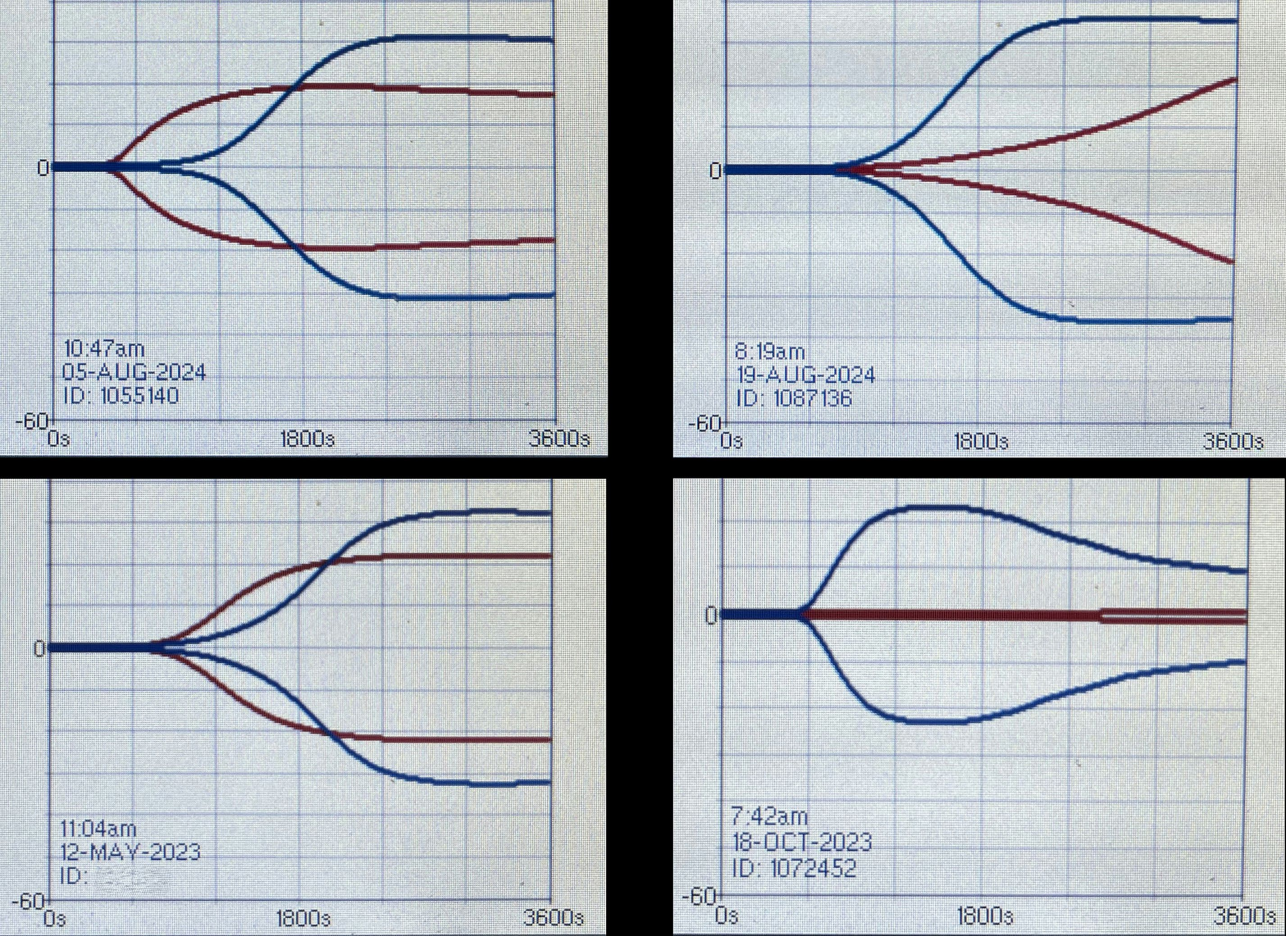
Viscoelastic tracings of equids envenomated in Colorado by prairie rattlesnakes (*Crotalus viridis viridis*) and obtained at admission (red tracing) and 12–24 h after antivenom administration (blue tracing). All tracings at admission supported hypocoagulability secondary to decreased propagation phase. Courtesy of Dr. Migliorisi, DVM, DACVIM (LAIM), DACVECC, Roaring Fork Equine Medical Center, USA.

**Figure 7:**
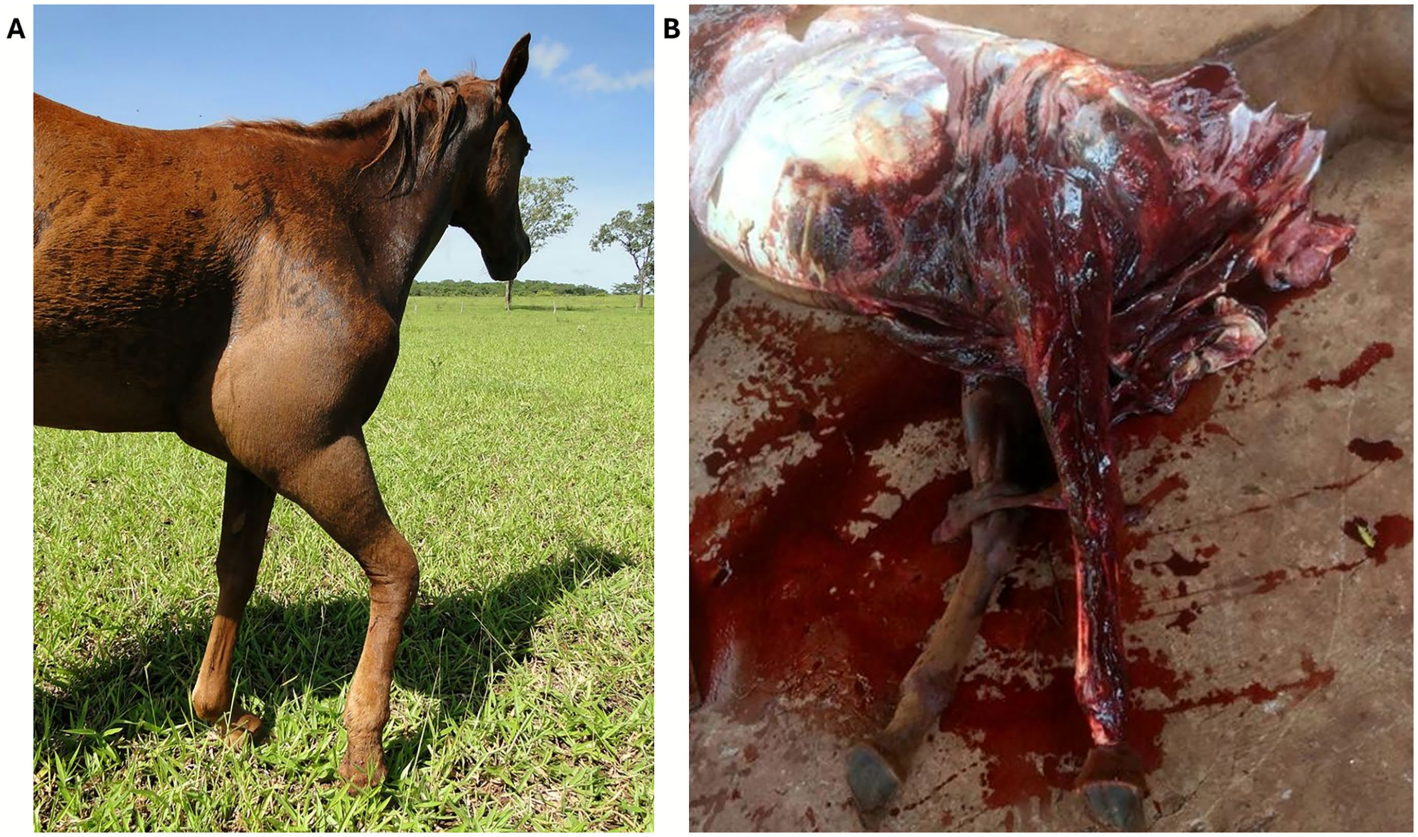
**(A)** *Bothrops jararaca* bite on a horse leg in Brazil. **(B)** Same horse at postmortem examination, showing the degree of tissue hemorrhage. Courtesy of Dr. Batista, MV, PhD, Universidad Federal de Mato Grosso Du Sol.

**Figure 8:**
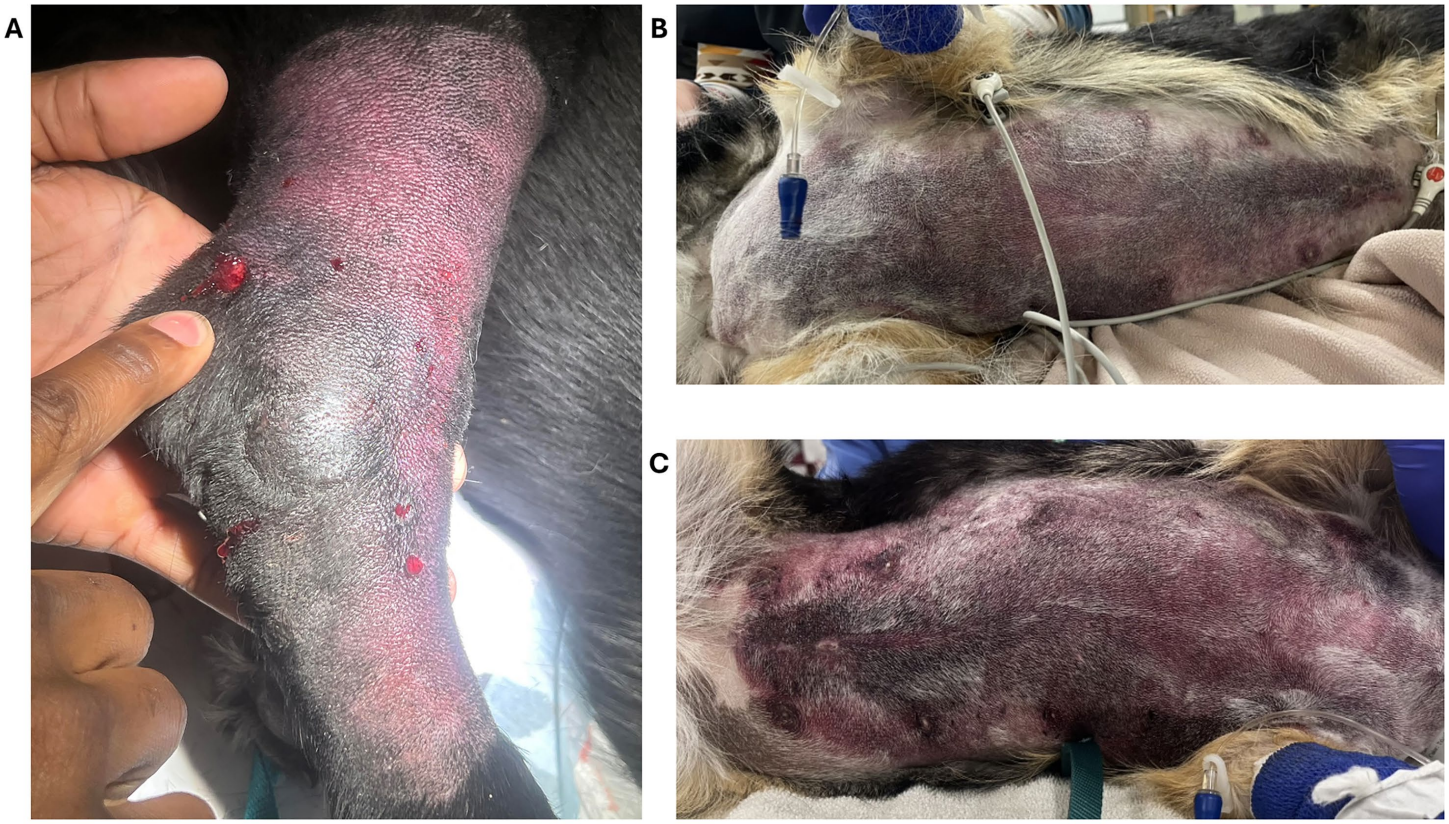
**(A)** Compartment syndrome on the right tarsus of a dog envenomated in Texas, presumably by Western diamondback rattlesnake (*Crotalus atrox*). Courtesy of Dr. Broussard, DVM, MT Venom, USA. **(B,C)** Diffuse ventral abdominal cutaneous ecchymosis in a Corgi envenomated in Colorado by a prairie rattlesnake (*Crotalus viridis viridis*). The dog eventually developed cellulitis and fasciitis. Courtesy of Dr. Rutten, DVM, DACVECC, Colorado State University, USA.

**Figure 9:**
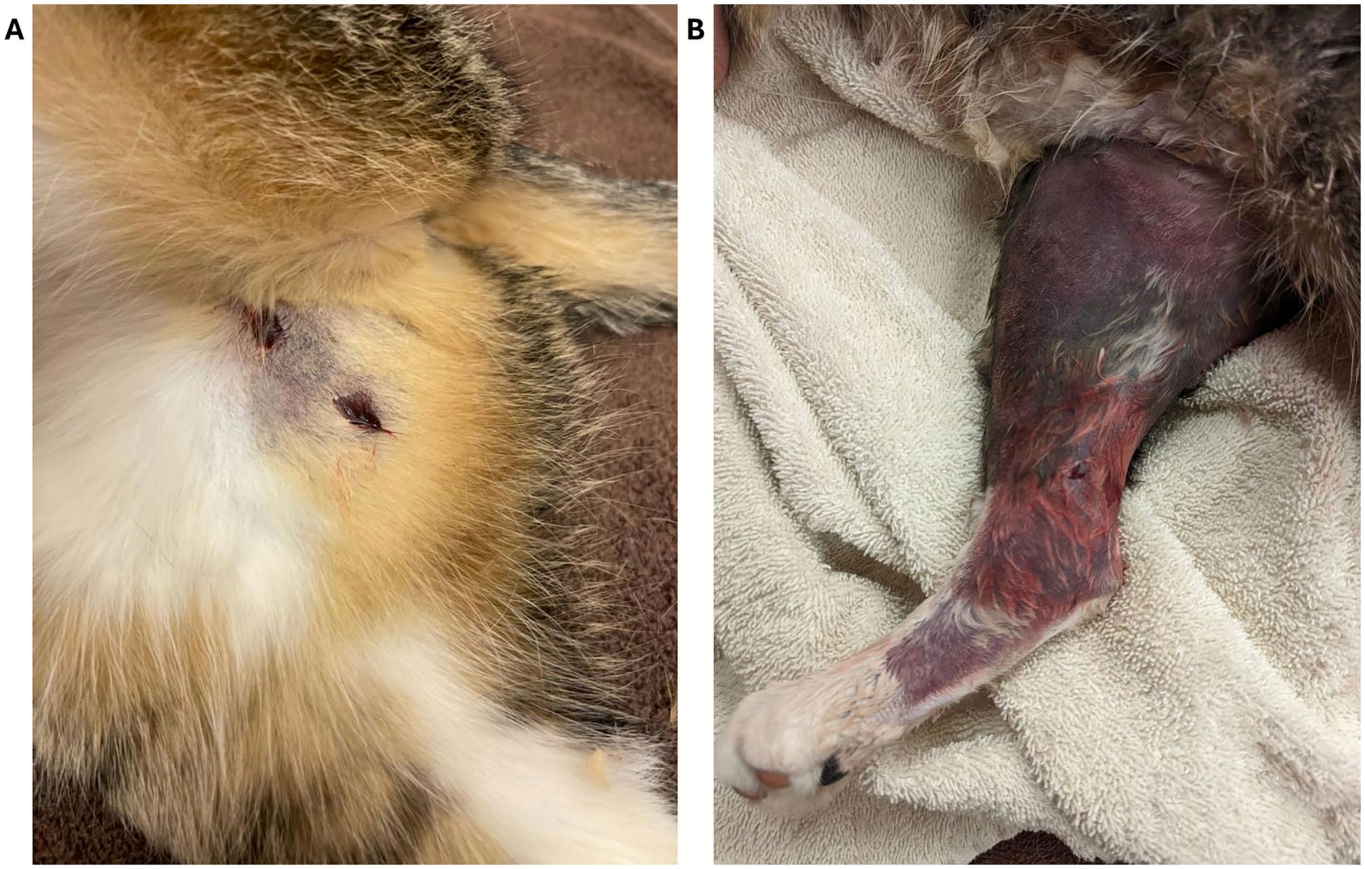
**(A)** Fang marks on the right thigh of a 3-year-old female cat envenomated in Southern California, presumably by a Southern Pacific rattlesnake (*Crotalus helleri*). Picture taken within 1 h of presentation. **(B)** Marked ecchymosis and compartment syndrome on the same cat on day 2. Courtesy of Dr. Broussard, DVM, MT Venom, USA.

In Australia, dogs bitten by tiger snakes (*Notechis scutatus*) often show marked depletion of clotting factors and prolonged PT/aPTT (82), while those envenomated by tiger or brown snakes (*Pseudonaja textilis*) typically have abnormal clotting times but normal platelet counts (83). In contrast, prairie rattlesnake (*Crotalus viridis viridis*) bites in Colorado are commonly associated with thrombocytopenia (84). Viscoelastic testing, such as thromboelastography, improves detection of hypocoagulability even when standard clotting times appear normal, as demonstrated in dogs from Southern California and Florida (85). Regional differences are also evident in South Africa (86), where puff adder bites cause longer reaction times on thromboelastography and lower platelet counts compared to cobra bites. Although Elapid envenomation generally causes fewer hemostatic abnormalities than Viperid bites, severe coagulopathy can still occur. Data in horses are limited (85–87), but Viperid bites (Figure 5) may cause spontaneous bleeding, thrombocytopenia, prolonged PT, aPTT and hypocoagulable viscoelastic tracings (Figure 6), while Elapid bites in Australia typically leave PT, aPTT, and platelet counts unchanged. Furthermore, evidence of hemorrhage in envenomated animals can often be found on post-mortem examination (Figures 2B, 7B) (86, 88, 89). Potential complications triggered by the disruption of hemostasis and concurrent capillary damage include diffuse ecchymosis, cellulitis and fasciitis (Figure 8), extravasation of blood resulting in compartment syndrome (Figure 9B), as well as life-threatening pulmonary embolism (90–93), cerebral infarction, and intracranial hemorrhage (88, 94).

## Snake envenomation in other domesticated species

**Figure 10:**
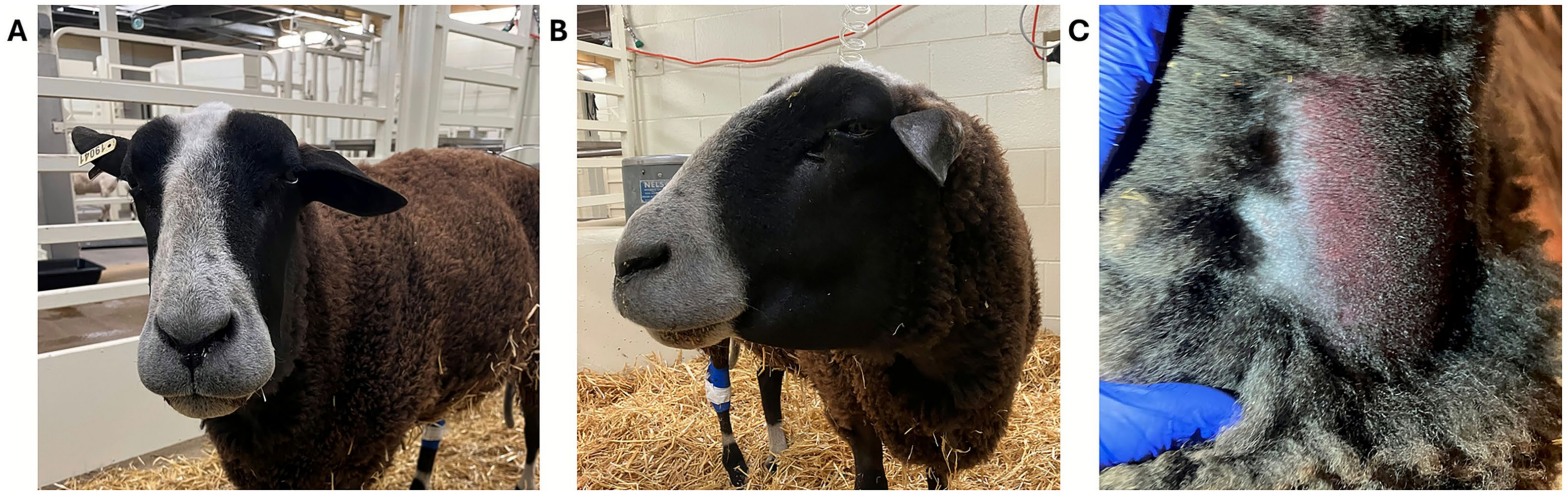
Muzzle snakebite on a sheep envenomated in Colorado by a prairie rattlesnake (*Crotalus viridis viridis*), and evidence of neck skin ecchymosis and discoloration. **(A)** Courtesy of Dr. Raabis, DVM, DACVIM, Colorado State University, USA. **(B,C)** Courtesy of Dr. Mullins, DVM, Kansas State University, USA.

**Figure 11:**
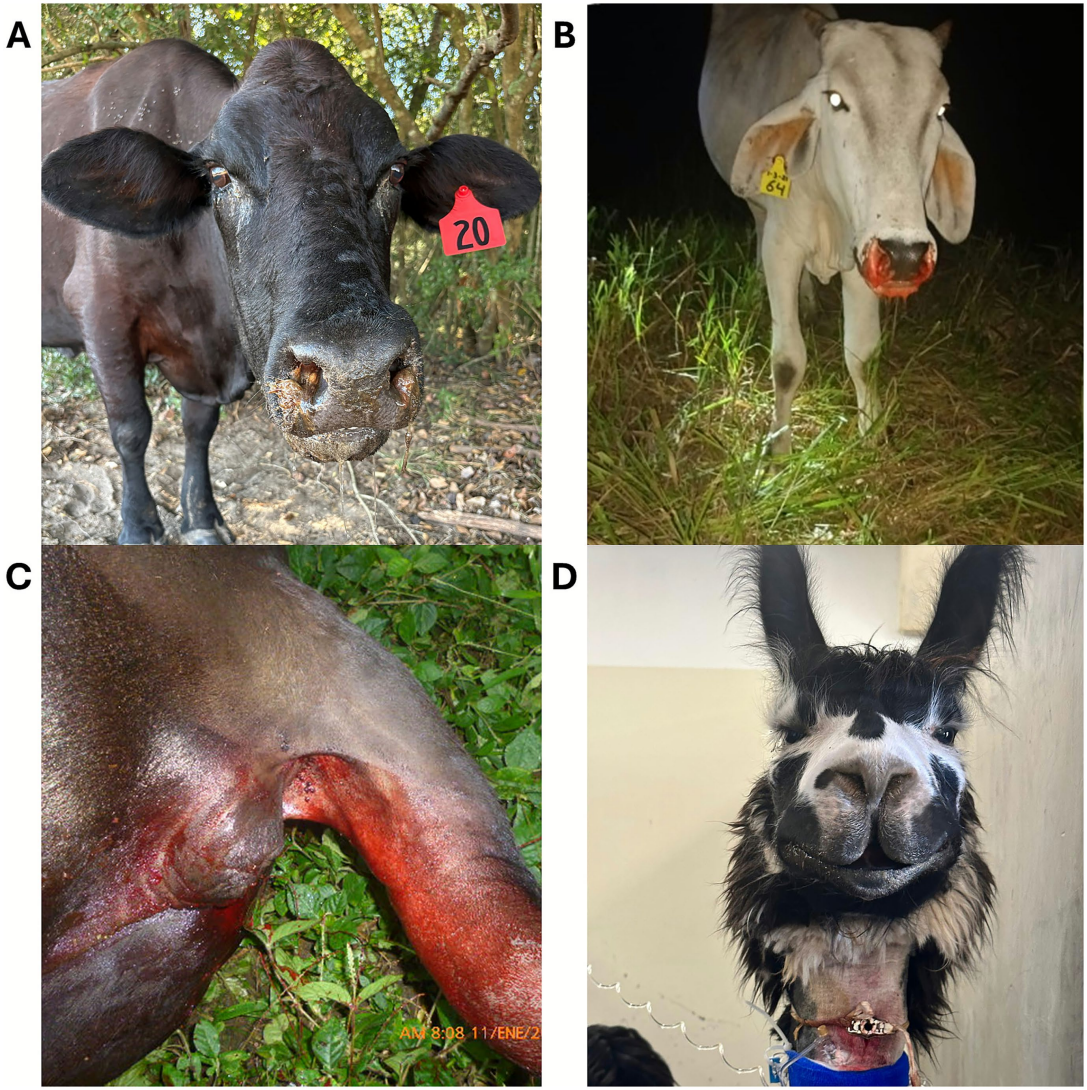
**(A)** Muzzle snakebite on a cow envenomated in Texas. Courtesy of Dr. Hobbs, DVM, DACVIM (LAIM), Texas A&M University, USA. **(B)** Epistaxis and **(C)** melena in cows envenomated by *Bothrops asper* (“fer-de-lance”) in Costa Rica. Courtesy of Instituto Clodomiro Picado, Costa Rica. **(D)** Llama envenomated on the muzzle in Northern California and which required a temporary tracheotomy. Courtesy of Dr. Quattrini, DVM, DACVIM (LAIM), DACVECC, University of Georgia, USA.

Snake envenomation in livestock species, such as small ruminants, camelids and cattle, is likely underreported in literature, although it has been known to occur (Figures 10, 11). Most available information comes from Central and South America. Cattle envenomated in Costa Rica by fer-de-lances (*Bothrops asper*), a Viperid snake, developed clinical signs that ranged from moderate to severe (95). Typical signs of Viperids envenomation, such as swelling and hemorrhage at the bite site, were reported, as well as lethargy and coagulopathy. Coagulopathy was manifested as bleeding from ears, nose, eyes, and evidence of blood in feces and urine. In more severe cases, pulmonary edema and death were recorded (95). These clinical manifestations are also consistent with a previous report on envenomated cattle in the same region (96). Cows envenomated in India developed swelling at the bite site, which was associated with respiratory distress when the bite occurred on the face, and coagulopathy manifested as epistaxis, hematochezia, melena, and hematuria (97). In the same study, laboratory evidence of coagulopathy included significant thrombocytopenia and increased clotting times. Snake envenomation in small ruminants (sheep and goats) has also been reported. In Brazil, sheep envenomated by *Bothrops* spp. snakes developed edema at the bite site followed by skin sloughing (98). Bleeding from eyes, ears and gingival mucosa developed in two sheep presumed to have been envenomated by *Bothrops* spp. snakes (99). Goats envenomated by Northern Pacific rattlesnakes (*C. oreganus*) in Northern California developed swelling at the bite site, respiratory distress, recumbency, facial nerve deficits and thrombocytopenia (100). Similar clinical signs of upper airway obstruction developed in two goats envenomated in Portugal resulting in asphyxiation (101). Elapid snake envenomation in cattle in Australia and snake envenomation in camels in Saudi Arabia has also been reported, however, without mention of clinical findings or treatments used (102, 103). Mortality from these studies appears quite variable, ranging from 0 to 100%, and is likely influenced by the snake species, time elapsed between bite recognition and initiation of treatment, and the type of treatment administered. Two studies in North America describe snake envenomation in New World camelids (104, 105). Common findings were respiratory distress, secondary to the snakebite located on the muzzle and upper airway obstruction, and thrombocytopenia. Hemolysis was a consistent finding in New World camelids envenomated by *C. viridis viridis* (prairie rattlesnake) in Colorado (105).

Captive and domesticated birds, just like other domesticated animal species, are also at risk of snakebites. Reports of avian envenomation are available for several species of birds and caused by Viperid, Elapid, Colubrid and Lamprophiid snakes (106). Reported clinical signs include swelling at the bite site, coagulopathies, ranging from mild hemorrhage at the bite site to ecchymoses and severe systemic hemorrhage, as well as neurological dysfunction such as flaccid paralysis, head droop and convulsions (106).

## Treatment of snakebite in veterinary medicine with focus on North America

The profound differences in venom composition across snake families and species, even when in similar geographical locations, are reflected by the diverse clinical pictures and severity faced at the time of patient’s evaluation. The reader is invited to familiarize themselves with the most common venomous snake species in their practicing area to identify possible patterns of clinical envenomation. A thorough list of venomous snake species present in different regions of United States is available (107).

**Figure 12:**
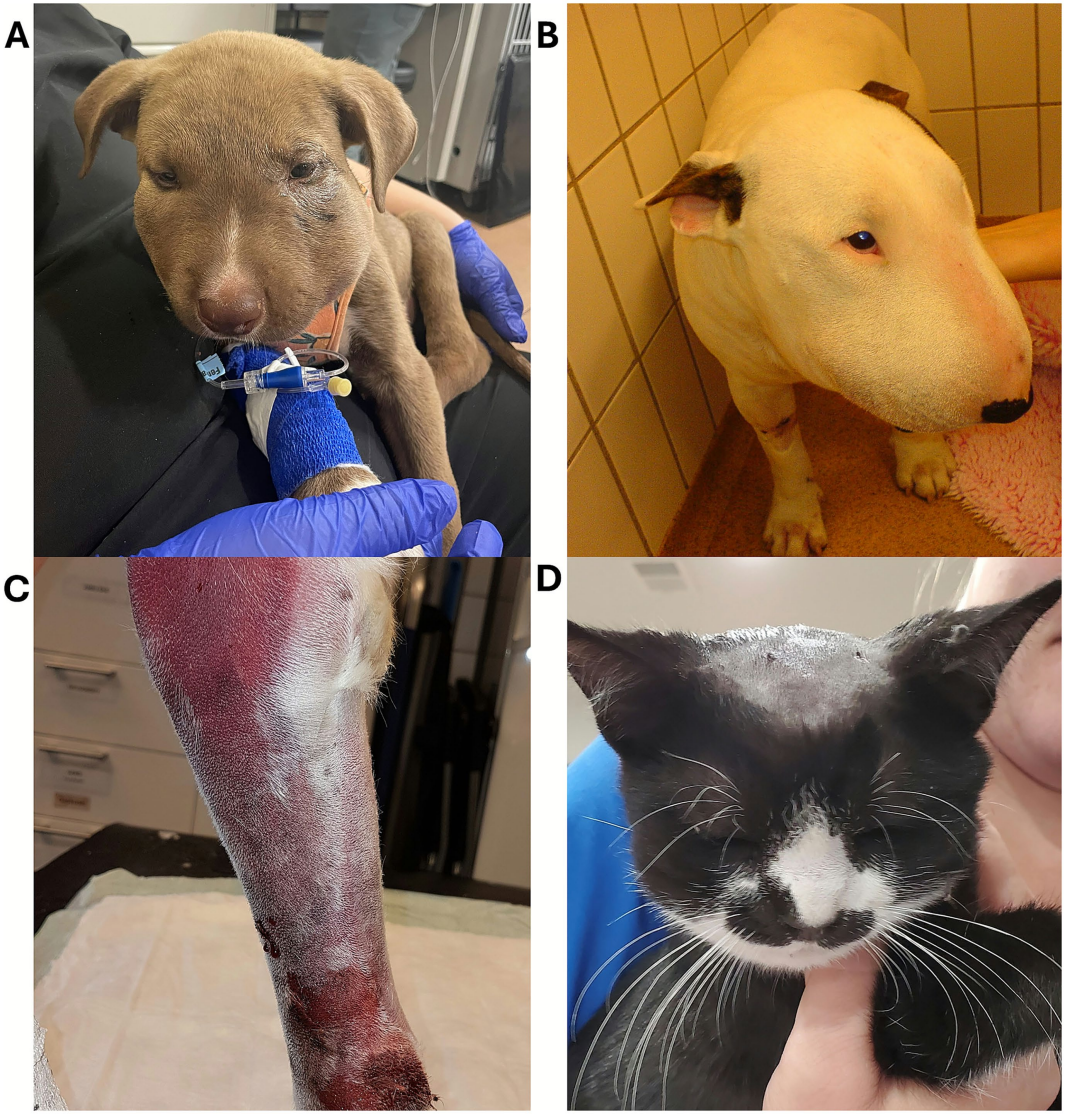
**(A)** Marked facial swelling in dogs envenomated by a prairie rattlesnake (*Crotalus viridis viridis*) in Colorado (courtesy of Dr. Talbot, BVSc, DACVECC, Colorado State University, USA) and **(B)** by a European adder (*Vipera berus*) (courtesy of Dr. Pelander, DVM, DECVIM-CA, Swedish University of Agricultural Sciences, Sweden). **(C)** Ecchymosis secondary to European adder (*Vipera berus*) bite on a dog’s leg (courtesy of Dr. H. Harjen, BVetMed, Norwegian University of Life Sciences, Norway). **(D)** Fang marks on the head of an envenomated cat in Colorado (courtesy of Dr. Darcy, DVM, Twin Forks Clinic, USA).

**Figure 13:**
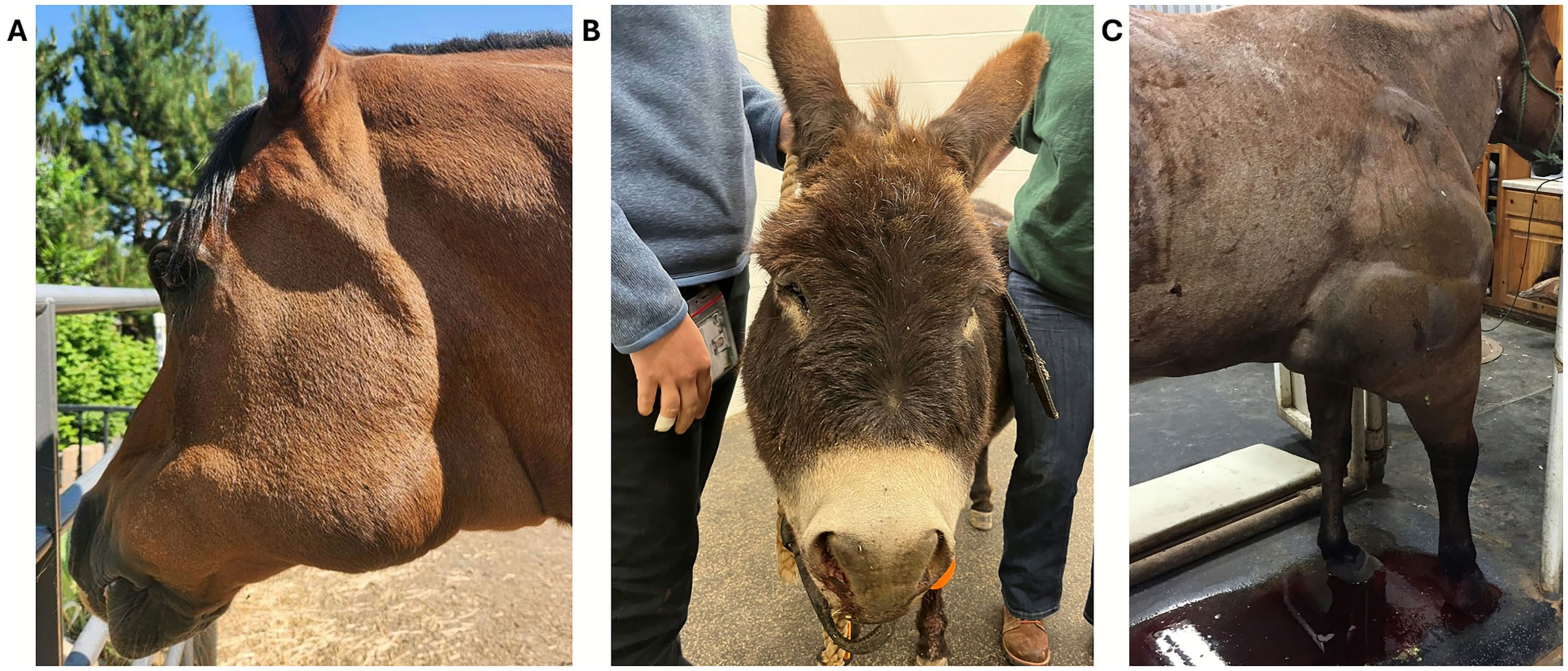
Throatlatch **(A)** and facial **(B)** swelling after prairie rattlesnake (*Crotalus viridis viridis*) envenomation in a horse and a donkey in Colorado. Courtesy of Dr. Migliorisi, DVM, DACVIM (LAIM), DACVECC, Roaring Fork Equine Medical Center, USA. **(C)** Marked swelling involving the distal to proximal right forelimb in a Quarter Horse bitten on the elbow region in Texas. Courtesy of Dr. Bevevino, DVM, DACVIM (LAIM), Roaring Fork Equine Medical Center, USA.

In North America most snakebites in veterinary species are caused by Viperids. Dogs are most frequently bitten on the head, followed by their legs and rest of the body (Figure 12). Cats are generally bitten on their front legs, and less frequently on their head or body (Figures 9, 12D). Likewise, snakebites in cattle most frequently occurred on the forelimbs, and less frequently on the head (97). Snakebites in New World camelids and horses generally occur on the muzzle, but bites on legs can also occur and sometimes causing marked swelling (Figures 11, 13). Because of the specific respiratory tract anatomy that makes horses obligated nasal breathers, the acutely envenomated horse is at its highest risk of death due to upper airway obstruction and asphyxiation secondary to the muzzle swelling resulting from the snakebites. Hence, the first line of treatment is based on ensuring airway patency. This can be achieved through the placement of plastic/rubber tubes (e.g., pieces of garden hose, old tracheal tubes) in the nasal passages up to the level of the medial canthus of the eye and secured at the nostrils with sutures or tape. However, resistance encountered when advancing the plastic/rubber tubes may be an indication of the need to perform a tracheotomy.

Following initial airway stabilization, intravenous access should be established for the administration of antivenom and fluid therapy when indicated. Antivenom is considered the gold-standard treatment for snake envenomation because it neutralizes venom toxins, therefore preventing and minimizing local and systemic adverse effects. Currently available antivenoms are obtained by collecting serum of animals, mostly large domesticated species such as horses and sheep, which have been immunized using venom toxins of snake species of interest (108). Timely administration of antivenom reduces the spread of swelling at the bite site, reverses coagulopathies and blocks progression of neuropathies. Currently, in the United States there are three veterinary- and two human-approved antivenom formulations (Table 2) and they all target Viperid snakes (i.e., genera *Crotalus*, *Agkistrodon*, *Sistrurus*). There are also several foreign antivenom products that can be imported with a special USDA permit and state-veterinarian approval, namely, the F(ab’)2 Antivipmyn (Polivalent Anti-snake Fabotherapic, Instituto Bioclon, Mexico City, Mexico) and the whole IgG PoliVet-ICP (Instituto Clodomiro Picado, San José, Costa Rica). Similarly, two foreign antivenoms designed for coral snake envenomation can be imported following the same process: the Coralmyn, a polyvalent F(ab’)2, equine-derived antivenom (Instituto Bioclon, Mexico City, Mexico) and the whole equine IgG Anticoral Antivenom (Instituto Clodomiro Picado Universidad De Costa Rica). Both the Coralmyn and Anticoral Antivenom are produced by immunizing horses with coral snakes indigenous to Mexico, Central America, and Colombia, although their efficacy against the venom of North American coral snakes has been demonstrated (21, 22, 109, 110). Whole IgG antivenoms are obtained by using an ammonium sulfate precipitation process. However, this precipitation process may be inefficient in eliminating α and β globulins, IgM, and protein aggregates, which may later increase the risk for acute adverse reactions, such as urticaria, fever, altered level of consciousness and anaphylaxis (111). The Fc region of IgG can also activate complement, contributing to immunogenicity; studies in dogs (112) report higher reaction rates with whole IgG products (113–115). Cleaving the Fc portion to produce F(ab’)₂ or Fab fragments reduces these effects and improves tissue penetration due to smaller molecular size (~100 kDa and ~50 kDa, respectively). However, fragment antivenoms are cleared faster, often requiring repeated dosing. Manufacturing processes, such as caprylic acid purification, further enhance purity and reduce reaction rates compared to ammonium sulfate alone. Preservatives like phenol and thimerosal may also increase risk, particularly with rapid undiluted administration (113, 116, 117).

**Table 2:** List of antivenom formulations commercially available in North America.

Brand name	Structure	Origin	Polyvalency	Preservatives	Notes
Antivenin Crotalid Polyvalent, APC (Boehringer Ingelheim, St. Joseph, MO)	Whole IgG	Equine	*Crotalus adamanteus* (Eastern diamondback rattlesnake), *Crotalus atrox* (Western diamondback rattlesnake), *Crotalus terrificus* (Central and South American rattlesnakes), *Bothrops atrox* (Fer-de-lance)	Phenol, thimerosal	Reconstitution may take up to 90 min. Labeled for dogs. Off-label use in new-world camelids.
Rattler Antivenom (Mg Biologics, Ames, IA)	Whole IgG	Equine	*Crotalus adamanteus* (Eastern diamondback rattlesnake), *Crotalus atrox* (Western diamondback rattlesnake), *Crotalus viridis viridis* (Prairie rattlesnake), *Crotalus scutulatus* type A (Mojave rattlesnake)	None	Frozen product. Half-life 10 days in dogs and cats, 21 days in horses. Labeled for horses, dogs and cats.
VenomVet (MT Venom LLC, Canoga Park, CA)	F(ab’)2	Equine	*Bothrops alternatus* (Urutu Lancehead), *Bothrops diporus* (Chaco Lancehead), *Crotalus durissus terrificus* (South American rattlesnake), *Crotalus simus* (Central American rattlesnake), *Bothrops asper* (Terciopelo)	Phenol	Refrigerated product. Half-life 2-4 days.Labeled for dogs. Off-label use in cats, horses, small ruminants. To be replaced by 2027 by VenomVet Plus.
VenomVet Plus (MT Venom LLC, Canoga Park, CA)	F(ab’)2	Equine	*Crotalus atrox* (Western diamondback rattlesnake), *Crotalus adamanteus* (Eastern diamondback rattlesnake), *Crotalus scutulatus* Type A (Mojave rattlesnake), *Crotalus viridis viridis* (Prairie rattlesnake), *Agkistrodum piscivorus* (Cottonmouth)	Phenol	Refrigerated product. Half-life 2-4 days. Labeled for dogs, cats and horses. USDA code 6101.05.
Anavip (Rare Disease Therapeutics, Inc., Franklin, TN)	F(ab’)2	Equine	*Bothrops asper* (Terciopelo), *Crotalus simus* (Central American rattlesnake), *Crotalus atrox* (Western diamondback rattlesnake), *Crotalus adamanteus* (Eastern diamondback rattlesnake), *Crotalus scutulatus* Type A (Mojave rattlesnake), *Agkistrodum piscivorus* (Cottonmouth), *Agkistrodon contortrix* (Copperhead)	None	Reconstitution takes few seconds. Half-life 5.5 days. Labeled for human use.
CroFab (BTG International Inc., Brentwood, TN)	Fab	Ovine	*Crotalus atrox* (Western diamondback rattlesnake), *Crotalus adamanteus* (Eastern diamondback rattlesnake), *Crotalus scutulatus* Type A (Mojave rattlesnake), *Agkistrodum piscivorus* (Cottonmouth)	None	Reconstitution takes less than 7 min. Half-life 4–24 h. Labeled for human use. Off-label use in horses, dogs and cats.

The faster clearance from the body of some antivenoms, as well as the low degree of peripheral tissues penetration of others, may increase the risk for development of recurrence phenomena. Recurrence phenomena are also known as recurrent venom antigenemia, and they indicate the development of a second wave of toxicity after the initial improvement associated with antivenom administration. There are several proposed mechanisms on why recurrence phenomena may develop, and more than one mechanism is likely to play a role. Recurrence phenomena could develop secondarily to: (1) a pharmacokinetic and pharmacodynamic venom/antivenom mismatch, (2) separation of venom/antivenom complex after the initial binding, (3) late onset of venom toxins previously trapped in swollen tissues, (4) development of host antivenom immune response (118, 119). In human patients this often manifests as late onset coagulopathy. Similarly, in dogs persistent or recurring coagulopathy has been reported (120, 121). In horses, common clinical manifestations of recurrence phenomena include peripheral neuropathy, myocardial damage, dysrhythmias, gastrointestinal dysfunction, and laminitis (64, 85, 89, 122–124). A previous study showed that horses developed their highest troponin peak concentration at 11 days after the envenomation event, highlighting the significant effects that venom toxins can have even days after the acute clinical signs (64).

Antivenom selection and dosing regimens for each of these products are influenced by the patient’s clinical status at the time of evaluation, the patient’s size, product availability, medical setting and cost. Patient’s clinical status can be determined using a snakebite severity score (SSS), and veterinary medicine versions are available specifically for rattlesnake bites in horses and generic snakebite in dogs (87, 125). Severity of clinical signs depends on a variety of snake and patient factors. When the snakebite does not result in the injection of venom, this is referred to as a dry bite, which is characterized by the lack of local swelling and systemic signs. Importantly, Elapid snake bites, compared to Viperids, normally do not cause any swelling at the venom injection site, which may delay or confound a final diagnosis. Severity of envenomation is directly correlated to the snake-size and inversely correlated to the victim’s size (126, 127). It is known that children are at higher risk of limb amputation compared to adults, possibly as a consequence of a relatively higher venom concentration in relation to their body size (128). Currently, only one veterinary medicine study reports the clinical presentation and antivenom vials used in a population of puppies, but due to the small number of cases included it remains difficult to draw final conclusions (129). Nevertheless, it is known that a smaller body size in dogs is associated with increased mortality (50). It is also hypothesized that miniature equids may develop more severe clinical signs compared to normal size horses due to the lower degree of venom dilution in their smaller body (124). Although some degree of cross-reactivity across antivenoms is possible, clinicians are encouraged to become familiar with the most common snake species present in their area and, when the information is available, with the variations in venom composition that occur within the same species. For example, prairie rattlesnake populations in the same state (Colorado) can exhibit a myotoxin-rich or metalloproteinase-rich phenotype venom solely based on their geographical location (130). Different species within the same genus can also significantly influence clinical severity and response to treatment after a snake bite. For instance, envenomated New World camelids from different states (California and Colorado) had a markedly different mortality rate even when treated with the same antivenom, thus suggesting an underlying different venom composition and potential need for more frequent antivenom re-dosing based on the geographical location and rattlesnake species (104, 105). Veterinarians should be familiar with the specific features and characteristics of antivenom products available in the market. For example, in case of Mojave rattlesnakes’ bite, or clinical signs consistent with this type of envenomation, an antivenom known to target this snake’s neurotoxins should be used. Available antivenoms have different guidelines for product handling. The ACP product requires reconstitution prior to being used, which can take up to 90 min. This becomes particularly relevant when the antivenom needs to be rapidly administered. The Rattler Antivenin comes as a frozen product, potentially limiting its use in an equine field practice setting. Both VenomVet Plus and CroFab require refrigeration, however, the VenomVet Plus comes in vials ready to be used, while the CroFab needs to be reconstituted, a process involving less than 7 min. Although Fab antivenom has been successfully used in horses, dogs and cats, the higher cost for each vial and the need for repeated dosing have limited its regular use in veterinary medicine (87, 131, 132).

Human guidelines for crotaline envenomation recommend the administration of 4–6 vials of Fab antivenom to a clinical patient (one with swelling, coagulopathy, systemic signs), followed by reassessment and redosing if necessary (133). Dosing recommendations in veterinary patients are purely anecdotal and often based on personal clinical experience, the patient’s clinical status and response to treatment. In companion animal medicine, additional confounding factors originate from the heterogeneity of available studies in regards to the antivenom product used, extremes of dog breed sizes compared to the more homogenous human patients, owner’s financial limitations impacting standards of care, and the retrospective nature of some studies (132, 134–136). Nevertheless, benefits from the use of antivenom are consistently reported in companion animals, such as a quicker resolution of bite-site edema (137). Two studies on equine envenomation in North America report the type and number of vials used (87, 124). Based on this limited data, and personal experience, it appears that 1–2 vials are generally able to counteract the effect of snake venom in most horses, based on the observed improvement in RBSS, laboratory variables, or both. Subsequent adjustments are made based on patient’s initial response. A prospective study involving envenomated horses in Arizona, California, Colorado, and South Dakota found that the administration of 1 vial of F(ab’)2 antivenom was adequate in more than 80% of cases in stabilizing the animal and leading to a positive outcome, based on a 4-week follow-up (138). The lack of improvement in bite site swelling, coagulation parameters, or both, likely indicate the need for a second unit of antivenom. Human guidelines recommend administering antivenom within 2–4 h of the snakebite (139). This is not always possible in veterinary medicine, particularly in horses, since the snake bite is often not witnessed and due to the time that elapses between owner’s recognition of the condition, field veterinary evaluation, and transportation to a referral facility. Nonetheless, the administration of antivenom to envenomated horses, outside of what is considered the optimal therapeutic window in people, seems to be effective in neutralizing venom toxins and ameliorate clinical and laboratory abnormalities. This should encourage equine clinicians to consider antivenom therapy in their patients, regardless of the number of hours that may have gone by since the envenomation.

Other treatments, apart from antivenom, should be considered following envenomation. Venom phospholipases cause progressive hydrolysis of cell membrane phospholipids, which are then used to generate pro-inflammatory mediators, driving a state of systemic inflammation. Therefore, non-steroidal anti-inflammatory drugs (NSAIDs) should be administered to dampen the inflammatory cascade, as well as providing a first line of analgesia. The use of NSAIDs is mostly accepted in horses, due to the side effects associated with other analgesics such as opioids. Guidelines in envenomated dogs do not recommend using NSAIDs due to the risk of nephropathy, gastrointestinal bleeding and the potential interference with platelet function (134). Currently, there is no evidence that the administration of flunixin meglumine to healthy horses impacts coagulation function (140). Presence of marked swelling at the bite site is associated with the development of compartment syndrome, a severely painful complication characterized by increased pressure within tissues. Consequently, if left untreated, secondary nerves, muscles and vessels damage can occur. Most envenomated animals will experience a certain degree of compartment syndrome, and this should be addressed and treated with a combination of antivenom and analgesics. Available analgesic options include intravenous NSAIDs, intramuscularly opioids, and topical diclofenac and lidocaine ointments. The oral route of administration should be avoided in cases of muzzle bites until the swelling resolves and the patient’s comfort improves. The sooner the local swelling resolves, the quicker the horse becomes able again to prehend food. Based on one of the authors’ clinical experience (A.M), appreciable improvement in swelling at the bite site in horses, most frequently the muzzle, becomes evident between 2 and 12 h after the antivenom administration. Importantly, the type of antivenom used may dictate the success in limiting further swelling spread. This is particularly true for whole IgG formulations which, due to their larger size, may be unable to penetrate the swollen tissues and neutralize venom metalloproteinases and hyaluronidase (141). Fasciotomy of the swollen areas is not recommended, and appropriate and adequate use of antivenom remains the treatment of choice to limit worsening of compartment syndrome. Ancillary treatments may include elevation of the affected area, if amenable, and laser therapy. Corticosteroids remain an equivocal treatment following snakebites. Those who advocate against their use cite the potential for increased susceptibility to infections, impaired tissue healing, risk of intestinal bleeding and increased cost. While supporters recognize several advantages including the improvement in local swelling, the cell-membrane stabilizing effect and the anti-inflammatory action. Although some clinical studies in envenomated people have shown a more rapid improvement in local swelling, other studies in companion animals failed to prove any benefit, with some reporting higher morality (50, 142–146). It is likely that the heterogeneity of results also depends on the herpetological differences across areas where the studies were performed, as well as on different study designs and drug protocols. Nevertheless, corticosteroids provide an upstream anti-inflammatory effect, which inhibits synthesis of tumor necrosis factor alpha (TNF-α), a known mediator of myocardial damage. Because TNF-α was shown to increase after envenomation, and to correlate with markers of myocardial injury and dysfunction, the use of corticosteroids may indeed have a role in the envenomated patient (64, 147, 148). Despite these apparent advantages, corticosteroids should not be used as a sole therapy after envenomation, but as an adjuvant to antivenom instead, which remains the mainstay of treatment. Additional treatments include IV fluid therapy if the animal is hypotensive, unable to drink due to muzzle swelling and when severe rhabdomyolysis/hemolysis is present, as this could evolve into life threatening kidney injury. Hypotension in the envenomated patient is likely multifactorial and related to bradykinin mediated vasodilation as well as loss of fluid through vomiting and hemorrhage. A myocardial depressing factor has been identified in the venom of Western diamondback rattlesnake (*Crotalus atrox*), which may contribute to the hypotension (149). Previous studies on envenomated horses in North America also mention blood transfusions due to hemolytic anemia in several animals (85, 87). It is important to note that most of the horses included in those studies did not receive antivenom, probably because of its unavailability at that time and the prohibitive cost of human products. Although hemolytic anemia remains a possible complication of snake envenomation in horses, the more regular use of antivenom may have reduced the incidence of life-threatening anemia requiring blood transfusions in this species. The use of fresh whole blood may be more relevant in companion animal medicine to treat severe anemia, coagulopathy and thrombocytopenia unresponsive to antivenom administration. However, blood transfusions could also worsen an underlying coagulopathy and the first line treatment for persistent coagulation disorders remains the co-administration of antivenom (134). Likewise, the administration of fresh frozen plasma is unlikely to correct an underlying deficiency of coagulation factors in the absence of antivenom (150). When facing persistent coagulopathies, the treatment of choice should be the administration of more antivenom to neutralize the toxins responsible for the observed hemostatic abnormalities (151). The use of antimicrobials post-envenomation has been a subject of debate for a long time. Bacterial flora in snake mouths is composed of a variety of Gram negative, Gram positive, and anaerobic bacteria, mostly derived from the intestinal content of preyed animals (152). Considering this, previous guidelines recommended the use of prophylactic broad-spectrum antimicrobials. Accordingly, studies in envenomated horses show a substantial use of antimicrobials ranging from beta-lactams, aminoglycosides, metronidazole, either as monotherapy or combined antimicrobials (85, 87). However, there is clear evidence from human and companion animal studies that the incidence of infections after envenomation is extremely low even when not using broad-spectrum antimicrobials (153, 154). In a recent study, despite none of the envenomated horses receiving broad-spectrum antimicrobials, there was no evidence of bite site (e.g., cellulitis, abscess formation) or systemic infection at the time of hospital discharge or at a 90-day follow-up (124). The low incidence of infection across species can be explained by the inherent antimicrobial properties of many peptides contained in snake venoms, particularly effective against the snake oral bacterial flora (155–157). Nevertheless, infections can still occur and identified risk factors in people include delay in medical care, inappropriate first-aid interventions (e.g., torniquets, tissue incisions, electroshock), tissue necrosis, rhabdomyolysis and thrombocytopenia (158, 159). Importantly, the typical signs of envenomation such as local swelling, pain, and serosanguineous discharge should not be interpreted as a sign of infection. Instead, continued monitoring for evidence of fever, new onset of swelling or purulent discharge should prompt collection of microbiologic samples for culture and susceptibility for a definite diagnosis and targeted treatment. Retention of snake teeth and fangs within tissues is also possible and could cause a foreign-body reaction (160). Similarly, tetanus vaccination status should be determined and boosters given if necessary. Mechanical ventilation is often required following Elapid snake envenomation due to development of respiratory muscle paralysis and hypoventilation (161). Dogs and cats mechanically ventilated for envenomation tend to have a good prognosis, with survival rates between 82% and approximately 92% (17, 162, 163). This is likely related to the nature of the underlying process which can be reversed with the use of antivenom.

The mortality rate in two previous studies on envenomated horses in North America was significantly higher than what is reported in humans and companion animals, reaching a 25% in the study by Dickinson et al. (85, 87). A more recent study, although small sized, reported a short and long-term mortality rate of 0% (124). It is plausible that the regular use of antivenom in more recent years explains this remarkable difference in survival. Survival rates in envenomated dogs in North America treated with antivenom approached 97% in one study (146). Similarly, 98% of envenomated dogs in South Korea survived to hospital discharge (164). An increasing time between recognition of the condition and hospitalization was recognized as a factor associated with poor outcome in some studies on dogs (84, 146). Among human patients, a subgroup of snakebite victims are pregnant women, which may survive to the snakebite but with perinatal death consequences. In a study in rural areas in Brazil, the rate of perinatal death (fetal and neonatal) was significantly higher in pregnant envenomated women (5.6%) compared to other non-envenomated pregnant women (2.6%) (165). A review of the scientific literature worldwide found an even higher case-fatality rate (19%) (166). It is thought the snake venom toxins may affect the fetus viability in several ways, for example by causing fetal anoxia due to maternal shock, by affecting the uterine muscle, by causing placental and uterine hemorrhage, or by directly affecting the fetus. Congenital fetal malformations are also reported following envenomation during pregnancy, both in women and laboratory animals (166–168). Available information in veterinary medicine is currently limited to two pregnant bitches envenomated in North America, both of which survived and delivered healthy puppies, and one pregnant cow in Turkey, who also survived, however, no information on subsequent calving was reported (169, 170).

## Innovative and translational therapies for snakebite envenomation

The understanding of venom biology and the antivenom manufacturing technology have significantly evolved and improved since the first experiments on antivenom by Henry Sewall and Albert Calmette (171, 172). Today, antivenom companies mostly use either immunized sheep or horses as donor animals. Nonetheless, ovine and equine-derived antivenoms come with a high operational cost, and an overall low yield when considering the millions of envenomation events in people worldwide. The need for accessible treatment solutions in impoverished rural countries continues to advance research, with the goal of developing alternatives to traditional antivenom formulations that could provide clinical efficacy, as well as higher product yields at lower operational costs (173). Bird (hens, ducks, ostriches) egg yolk antibodies (IgY) have been extensively researched in experimental settings for the neutralization of different types of toxins, among these snake venom toxins (174–178). The IgY technology offers many advantages, such as the lower operation cost per year compared to sheep and horses, the smaller amount of venom antigens needed to immunize avians, the high yield of eggs (~300 per hen each year) and antibodies (100–150 mg of IgY per 15 mL/yolk vs. 200 mg of IgG/bleed), their intrinsic inability to bind mammalian complement and Fc receptors, the noninvasive method of extraction from egg yolk, and the improvement in donor animal’s welfare compared to the stress and harm associated with blood collection from mammalian species (175). Despite the promising efficacy of IgY antivenoms, these remain restricted to in-vitro and laboratory animal models, mainly due to the lack of standardized production and regulatory approval for human injection and clinical trials. Recombinant technology has been used to create in-vitro plant-derived antibodies with antivenom properties (179–181). Plant derived antibodies are called pluribodies and, although they may provide sustainable antivenom production, they also present several flaws, such as their limited efficacy in neutralizing complex venoms. Camelids and some sharks produce unique antibodies called heavy-chain-only antibodies (HCAbs), in which the antigen recognition portion (i.e., variable domain) is termed VHH (variable heavy domain of heavy chain) (182, 183). The single-domain VHH fragments of HCAbs have been called nanobodies and present many advantages such as the small size (15–20 kDa), the ability to bind their target with extreme affinity and precision, the structural stability in broad ranges of pH and temperature, the low immunogenicity, and the ability to bind epitopes that are otherwise too small for conventional antibodies (184). Additionally, they can be easily expressed by bacterial systems. This recombinant technology has been used to produce nanobodies capable of neutralizing a variety of snake venom toxins, with encouraging results in laboratory settings (185–188). Most recently, researchers have been able to build ex-novo antivenom peptides capable of displacing venom neurotoxins already bound to their target receptors (189). These proteins, expressed through recombination by *E. coli* colonies, showed efficacy both *in-vitro* and *in-vivo* on laboratory animals. Small molecular therapeutics (SMTs) have recently gained attention as an adjunctive and early treatment in envenomated patients. Their use is not intended to replace antivenom, but to stabilize the patient prior to antivenom administration. Examples of SMTs include the PLA_2_ inhibitor varespladib, and the SVMPs inhibitors batimastat and marimastat (190, 191). Varespladib has been used in clinical snake envenomation, both human and veterinary, and has shown promising results (192, 193). Carbon monoxide releasing molecules (CORMs) are another SMT studied for their ability to stabilize and reverse hyperfibrinolysis caused by crotaline venom. The use of CORMs has shown promise in plasma samples collected from naturally envenomated patients when assessing TEG parameters associated with fibrinolysis (194). Another strategy to mitigate the effects of venom toxins is based not on their neutralization but on their removal from circulation using extracorporeal therapies. This approach has been successfully used in laboratory animals and human patients envenomated by different types of snakes and treated with therapeutic plasma exchange, hemodialysis or hemadsorption (195–200). In a controlled study involving rats envenomated with South American rattlesnake venom, 100% survival was observed in the hemoperfusion-treated group and the antivenom treated group, while all envenomated but untreated animals developed neurotoxicity and succumbed to envenomation. Early clinical applications in veterinary and human settings have demonstrated hemoperfusion and total plasma exchanges potential to reduce systemic toxin burden, mitigate organ damage, and improve survival in cases of severe envenomation (199). Collectively, these emerging therapies ranging from computationally designed proteins and synthetic antibodies to extracorporeal detoxification represent a paradigm shift toward safer, more effective, and globally accessible treatments for snakebite envenomation.

## Conclusion

Snake envenomation is a leading cause of mortality and high hospitalization costs in both human and veterinary patients worldwide. Despite recent attention in the literature to novel treatments for snake envenomation in both human and veterinary medicine, antivenom administration and supportive care are still the mainstays of treatment. Although small molecular therapeutics and treatments such as total plasma exchange and hemoperfusion hold promise as a potential treatment strategy for snake envenomation, significant challenges remain, such as individual patient immune responses, timely intervention, high cost and translating preclinical findings into effective therapies for envenomation patients.

## Glossary

3FTX: Three finger neurotoxin
AKI: Acute kidney injury
ALT: Alanine aminotransferase
AST: aspartate aminotransferase
aPTT: activated partial thromboplastin time
CLPs: C-type lectin-like proteins
DIC: Disseminated intravascular coagulation
ECM: Extracellular matrix
GLDH: Glutamate dehydrogenase
LAAO: L-amino acid oxidase
LDH: Lactate dehydrogenase
NSAIDs: Nonsteroidal anti-inflammatory drugs
PLA_2_: Phospholipase A_2_
PT: Prothrombin time
SMTs: Small molecular therapeutics
SVMPs: Snake venom metalloproteinases
SVSP: Snake venom serine proteases
TEG: Thromboelastography
TNF-α: Tumor necrosis factor α
VICC: Venom induced consumptive coagulopathy

## References

[1] World Health Organization. Snakebite envenoming 2023. Available online at: https://www.who.int/news-room/fact-sheets/detail/snakebite-envenoming (Accessed October 1, 2025).

[2] Bolon I Finat M Herrera M Nickerson A Grace D Schutte S . Snakebite in domestic animals: first global scoping review. Prev Vet Med. (2019) 170:104729. doi: 10.1016/j.prevetmed.2019.104729, 31421490

[3] Many copperhead snake bites require antivenom. Here’s how much that’ll cost you. Available online at: https://www.newsobserver.com/news/local/article262240987.html#storylink=cpy (Accessed October 1, 2025).

[4] Herzel B Batavia N Gavaza P Phan T Samones E Ruha AM . The cost of antivenom: a cost minimization study using the north American snakebite registry. J Med Toxicol. (2025) 21:320–6. doi: 10.1007/s13181-025-01072-x, 40227519 PMC12205100

[5] Tirosh-Levy S Solomovich-Manor R Comte J Nissan I Sutton GA Gabay A . Daboia (Vipera) palaestinae envenomation in 123 horses: treatment and efficacy of antivenom administration. Toxins (Basel). (2019) 11:168. doi: 10.3390/toxins11030168, 30893807 PMC6468471

[6] Chippaux JP Williams V White J. Snake venom variability: methods of study, results and interpretation. Toxicon. (1991) 29:1279–303. doi: 10.1016/0041-0101(91)90116-9, 1814005

[7] Tasoulis T Isbister GK. A review and database of Snake venom proteomes. Toxins. (2017) 9:290. doi: 10.3390/toxins9090290, 28927001 PMC5618223

[8] Kini RM Doley R. Structure, function and evolution of three-finger toxins: mini proteins with multiple targets. Toxicon. (2010) 56:855–67. doi: 10.1016/j.toxicon.2010.07.010, 20670641

[9] Dufton MJ Hider RC. Structure and pharmacology of elapid cytotoxins. Pharmacol Ther. (1988) 36:1–40. doi: 10.1016/0163-7258(88)90111-8, 3277206

[10] Feofanov AV Sharonov GV Dubinnyi MA Astapova MV Kudelina IA Dubovskii PV . Comparative study of structure and activity of cytotoxins from venom of the cobras *Naja oxiana*, Naja kaouthia, and *Naja haje*. Biochem Mosc. (2004) 69:1148–57. doi: 10.1023/b:biry.0000046890.46901.7e, 15527416

[11] Tilbury C. Observations on the bite of the Mozambique spitting cobra (*Naja mossambica* mossambica). S Afr Med J. (1982) 61:308–13. 7058469

[12] Scott EM Schlesener BN Shaw GC Teixeira LBC. Canine ocular and periocular snakebites requiring enucleation: a report of 19 cases. Vet Ophthalmol. (2019) 22:666–73. doi: 10.1111/vop.12638, 30716186

[13] Harvey AL. Cardiotoxins from cobra venoms: possible mechanisms of action. J Toxicol Toxin Rev. (1985) 4:41–69.

[14] Harvey A. Cytolytic toxins. In: Shier WT Mebs D, editors. Handook of toxicology. New York: Dekker (1990)

[15] Harvey A. Handbook of natural toxins. New York: Marcel Dekker 1991;5:85–106.

[16] Du XY Clemetson KJ. Snake venom L-amino acid oxidases. Toxicon. (2002) 40:659–65. doi: 10.1016/s0041-0101(02)00102-2, 12175601

[17] Campos S Allen-Durrance AE Schaer M Lynch A. Retrospective evaluation of *Micrurus fulvius* (eastern coral snake) envenomation and the use of mechanical ventilation in dogs and a cat (2011-2016): 8 cases. J Vet Emerg Crit Care (San Antonio). (2019) 29:662–7. doi: 10.1111/vec.12892, 31625672

[18] Chrisman CL Hopkins AL Ford SL Meeks JC. Acute, flaccid quadriplegia in three cats with suspected coral snake envenomation. J Am Anim Hosp Assoc. (1996) 32:343–9. doi: 10.5326/15473317-32-4-343, 8784725

[19] Kremer KA Schaer M. Coral snake (*Micrurus Fulvius Fulvius*) envenomation in five dogs: present and earlier findings. J Vet Emerg Crit Care. (1995) 5:9–15. doi: 10.1111/j.1476-4431.1995.tb00022.x

[20] Heinz JA Mankin J Pashmakova M. Transient megaesophagus following coral snake envenomation in three dogs (2013–2018). J Am Anim Hosp Assoc. (2020) 56:320. doi: 10.5326/jaaha-ms-6915, 33113557

[21] Perez ML Fox K Schaer M. A retrospective evaluation of coral snake envenomation in dogs and cats: 20 cases (1996-2011). J Vet Emerg Crit Care (San Antonio). (2012) 22:682–9. doi: 10.1111/j.1476-4431.2012.00806.x, 23153051

[22] Sullivan JM Aasen TL Fisher CJ Schaer M. Retrospective evaluation of clinical and clinicopathologic findings, case management, and outcome for dogs and cats exposed to *Micrurus fulvius* (eastern coral snake): 92 cases (2021-2022). Toxins (Basel). (2024) 16:246. doi: 10.3390/toxins16060246, 38922141 PMC11209501

[23] Mourier G Salinas M Kessler P Stura EA Leblanc M Tepshi L . Mambalgin-1 pain-relieving peptide, stepwise solid-phase synthesis, crystal structure, and functional domain for acid-sensing ion channel 1a inhibition. J Biol Chem. (2016) 291:2616–29. doi: 10.1074/jbc.M115.702373, 26680001 PMC4742732

[24] Reid PF. Alpha-cobratoxin as a possible therapy for multiple sclerosis: a review of the literature leading to its development for this application. Crit Rev Immunol. (2007) 27:291–302. doi: 10.1615/critrevimmunol.v27.i4.10, 18197810

[25] Fox JW Serrano SM. Timeline of key events in snake venom metalloproteinase research. J Proteome. (2009) 72:200–9. doi: 10.1016/j.jprot.2009.01.015, 19344655

[26] Fox JW Serrano SM. Insights into and speculations about snake venom metalloproteinase (SVMP) synthesis, folding and disulfide bond formation and their contribution to venom complexity. FEBS J. (2008) 275:3016–30. doi: 10.1111/j.1742-4658.2008.06466.x, 18479462

[27] Retzios AD Markland FS. Fibrinolytic enzymes from the venoms of *Agkistrodon contortrix contortrix* and *Crotalus basiliscus basiliscus*: cleavage site specificity towards the alpha-chain of fibrin. Thromb Res. (1994) 74:355–67. 8085237 10.1016/0049-3848(94)90151-1

[28] Shannon JD Baramova EN Bjarnason J Fox J. Amino acid sequence of a *Crotalus atrox* venom metalloproteinase which cleaves type IV collagen and gelatin. J Biol Chem. (1989) 264:11575–83. doi: 10.1016/s0021-9258(18)80102-8, 2745407

[29] Takeya H Arakawa M Miyata T Iwanaga S Omori-Satoh T. Primary structure of H2-proteinase, a non-hemorrhagic metalloproteinase, isolated from the venom of the Habu snake, *Trimeresurus flavoviridis*. J Biochem. (1989) 106:151–7. doi: 10.1093/oxfordjournals.jbchem.a122805, 2777746

[30] Lazarovici P Marcinkiewicz C Lelkes PI. From snake venom’s disintegrins and C-type lectins to anti-platelet drugs. Toxins. (2019) 11:303. doi: 10.3390/toxins11050303, 31137917 PMC6563238

[31] Kini RM. Excitement ahead: structure, function and mechanism of snake venom phospholipase A2 enzymes. Toxicon. (2003) 42:827–40. doi: 10.1016/j.toxicon.2003.11.002, 15019485

[32] Kini RM Evans HJ. A model to explain the pharmacological effects of snake venom phospholipases A2. Toxicon. (1989) 27:613–35. doi: 10.1016/0041-0101(89)90013-5, 2665186

[33] Black AR Donegan CM Denny BJ Dolly JO. Solubilization and physical characterization of acceptors for dendrotoxin and. beta.-bungarotoxin from synaptic membranes of rat brain. Biochemistry. (1988) 27:6814–20. doi: 10.1021/bi00418a025, 3196683

[34] Kirkpatrick LL Matzuk MM D'Nette CD Perin MS. Biochemical interactions of the neuronal pentraxins: neuronal pentraxin (NP) receptor binds to taipoxin and taipoxin-associated calcium-binding protein 49 via NP1 and NP2. J Biol Chem. (2000) 275:17786–92. 10748068 10.1074/jbc.M002254200

[35] Lambeau G Ancian P Barhanin J Beiboer S Nicolas J Verheij H . A family of receptors for venom phospholipases A 2. Toxicon. (1997) 35:474.

[36] Kerns RT Kini RM Stefansson S Evans HJ. Targeting of venom phospholipases: the strongly anticoagulant phospholipase A2 from *Naja nigricollis* venom binds to coagulation factor Xa to inhibit the prothrombinase complex. Arch Biochem Biophys. (1999) 369:107–13. doi: 10.1006/abbi.1999.1345, 10462445

[37] Serrano SM. The long road of research on snake venom serine proteinases. Toxicon. (2013) 62:19–26. doi: 10.1016/j.toxicon.2012.09.003, 23010164

[38] Khan SU Al-Saleh SS. Biochemical characterization of a factor X activator protein purified from *Walterinnesia aegyptia* venom. Blood Coagul Fibrinolysis. (2015) 26:772–7. 26407136 10.1097/MBC.0000000000000336

[39] Kitano ES Garcia TC Menezes MC Tashima AK Zelanis A Serrano SM. Cotiarinase is a novel prothrombin activator from the venom of *Bothrops cotiara*. Biochimie. (2013) 95:1655–9. doi: 10.1016/j.biochi.2013.04.006, 23619704

[40] Mukherjee AK. The pro-coagulant fibrinogenolytic serine protease isoenzymes purified from *Daboia russelii russelii* venom coagulate the blood through factor V activation: role of glycosylation on enzymatic activity. PLoS One. (2014) 9:e86823. doi: 10.1371/journal.pone.0086823, 24520323 PMC3919717

[41] Murakami MT Arni RK. Crystallization and preliminary X-ray crystallographic studies of Protac®, a commercial protein C activator isolated from *Agkistrodon contortrix contortrix* venom. Biochim Biophys Acta (BBA). (2005) 1752:202–4. doi: 10.1016/j.bbapap.2005.08.003, 16129664

[42] Sanchez EF Felicori LF Chavez-Olortegui C Magalhaes HB Hermogenes AL Diniz MV . Biochemical characterization and molecular cloning of a plasminogen activator proteinase (LV-PA) from bushmaster snake venom. Biochim Biophys Acta (BBA). (2006) 1760:1762–71. doi: 10.1016/j.bbagen.2006.08.023, 17034951

[43] Ullah A Masood R Ali I Ullah K Ali H Akbar H . Thrombin-like enzymes from snake venom: structural characterization and mechanism of action. Int J Biol Macromol. (2018) 114:788–811. doi: 10.1016/j.ijbiomac.2018.03.164, 29604354

[44] Zhang Y Xu W Ma B Huang K Song M Zhang N . Isolation and characterisation of a kallikrein-like enzyme from Agkistrodon halys pallas snake venom. J Sci Food Agric. (2012) 92:1497–503. doi: 10.1002/jsfa.4733, 22162083

[45] Felicori LF Souza CT Velarde DT Magalhaes A Almeida AP Figueiredo S . Kallikrein-like proteinase from bushmaster snake venom. Protein Expr Purif. (2003) 30:32–42. doi: 10.1016/s1046-5928(03)00053-6, 12821319

[46] Hung C-C Chiou S-H. Fibrinogenolytic proteases isolated from the snake venom of Taiwan habu: serine proteases with kallikrein-like and angiotensin-degrading activities. Biochem Biophys Res Commun. (2001) 281:1012–8. doi: 10.1006/bbrc.2001.4452, 11237764

[47] Vaiyapuri S Harrison RA Bicknell AB Gibbins JM Hutchinson G. Purification and functional characterisation of rhinocerase, a novel serine protease from the venom of *Bitis gabonica rhinoceros*. PLoS One. (2010) 5:e9687. doi: 10.1371/journal.pone.0009687, 20300193 PMC2837349

[48] Oliveira AL Viegas MF da Silva SL Soares AM Ramos MJ Fernandes PA. The chemistry of snake venom and its medicinal potential. Nat Rev Chem. (2022) 6:451–69. doi: 10.1038/s41570-022-00393-7, 35702592 PMC9185726

[49] Aroch I Harrus S. Retrospective study of the epidemiological, clinical, haematological and biochemical findings in 109 dogs poisoned by *Vipera xanthina* palestinae. Vet Rec. (1999) 144:532–5. doi: 10.1136/vr.144.19.532, 10378282

[50] Segev G Shipov A Klement E Harrus S Kass P Aroch I. *Vipera palaestinae* envenomation in 327 dogs: a retrospective cohort study and analysis of risk factors for mortality. Toxicon. (2004) 43:691–9. doi: 10.1016/j.toxicon.2004.03.001, 15109890

[51] Liapis K Charitaki E Psaroulaki A. Case report: spherocytic hemolytic anemia after envenomation by long-nosed viper (*Vipera ammodytes*). Am J Trop Med Hyg. (2019) 101:1442–5. doi: 10.4269/ajtmh.19-0611, 31674297 PMC6896858

[52] Chugh K. Snake-bite-induced acute renal failure in India. Kidney Int. (1989) 35:891–907. doi: 10.1038/ki.1989.70, 2651763

[53] Cobcroft RG Williams A Cook D Williams DJ Masci P. Hemolytic uremic syndrome following taipan envenomation with response to plasmapheresis. Pathology. (1997) 29:399–402. doi: 10.1080/00313029700169385 9423222

[54] Isbister G Little M Cull G McCoubrie D Lawton P Szabo F . Thrombotic microangiopathy from Australian brown snake (*Pseudonaja*) envenoming. Intern Med J. (2007) 37:523–8. doi: 10.1111/j.1445-5994.2007.01407.x, 17640187

[55] Leisewitz AL Blaylock RS Kettner F Goodhead A Goddard A Schoeman JP. The diagnosis and management of snakebite in dogs--a southern African perspective. J S Afr Vet Assoc. (2004) 75:7–13. 15214688 10.4102/jsava.v75i1.441

[56] Michal MT Eran L. Suspected *Vipera palaestinae* envenomation in three cats. Vet Hum Toxicol. (1999) 41:145–8. 10349702

[57] Brown DE Meyer DJ Wingfield WE Walton RM. Echinocytosis associated with rattlesnake envenomation in dogs. Vet Pathol. (1994) 31:654–7. doi: 10.1177/030098589403100604, 7863580

[58] Kim D Kim S Kim JK Lim JH Choi G Bae S . Clinical features and management of snake bites in 70 dogs in Korea. J Vet Sci. (2022) 23:e81. doi: 10.4142/jvs.22105, 36259100 PMC9715381

[59] Perry M Torres S Wells R Olver C Stewart S Granfone M. In-vitro exposure of feline red blood cells to rattlesnake venom causes echinocytosis. Toxicon. (2024) 248:108054. doi: 10.1016/j.toxicon.2024.108054, 39089489

[60] Walton RM Brown DE Hamar DW Meador VP Horn JW Thrall MA. Mechanisms of echinocytosis induced by *Crotalus atrox* venom. Vet Pathol. (1997) 34:442–9. doi: 10.1177/030098589703400508 9381655

[61] Masserdotti C. Unusual "erythroid loops" in canine blood smears after viper-bite envenomation. Vet Clin Pathol. (2009) 38:321–5. doi: 10.1111/j.1939-165X.2009.00145.x, 19392755

[62] Asmari AK Khan HA Banah FA Buraidi AA Manthiri RA. Serum biomarkers for acute hepatotoxicity of *Echis pyramidum* snake venom in rats. Int J Clin Exp Med. (2015) 8:1376–80. 25785140 PMC4358595

[63] Lervik JB Lilliehook I Frendin JH. Clinical and biochemical changes in 53 Swedish dogs bitten by the European adder--*Vipera berus*. Acta Vet Scand. (2010) 52:26. doi: 10.1186/1751-0147-52-26, 20416040 PMC2873270

[64] Gilliam LL Holbrook TC Ownby CL McFarlane D Sleeper MM Martin S . Cardiotoxicity, inflammation, and immune response after rattlesnake envenomation in the horse. J Vet Intern Med. (2012) 26:1457–63. doi: 10.1111/j.1939-1676.2012.01022.x, 23113840

[65] Harjen HJ Bjelland AA Harris J Gron TK Anfinsen KP Moldal ER . Ambulatory electrocardiography and serum cardiac troponin I measurement in 21 dogs envenomated by the European adder (*Vipera berus*). J Vet Intern Med. (2020) 34:1369–78. doi: 10.1111/jvim.15817, 32557821 PMC7379007

[66] Hoffman A Levi O Orgad U Nyska A. Myocarditis following envenoming with *Vipera palaestinae* in two horses. Toxicon. (1993) 31:1623–8. doi: 10.1016/0041-0101(93)90347-l, 8146876

[67] Pelander L Ljungvall I Haggstrom J. Myocardial cell damage in 24 dogs bitten by the common European viper (*Vipera berus*). Vet Rec. (2010) 166:687–90. doi: 10.1136/vr.b4817, 20511652

[68] Martinez J Londono LA Schaer M. Retrospective evaluation of acute kidney injury in dogs with pit viper envenomation (2008-2017): 56 cases. J Vet Emerg Crit Care (San Antonio). (2020) 30:698–705. doi: 10.1111/vec.13007, 32975046

[69] Braga JRM Jorge ARC Marinho AD Silveira JAM Nogueira-Junior FA Valle MB . Renal effects of venoms of Mexican coral snakes Micrurus browni and *Micrurus laticollaris*. Toxicon. (2020) 181:45–52. doi: 10.1016/j.toxicon.2020.04.095, 32339535

[70] Adhikari R Suriyagoda L Premarathna A De Silva N Dangolla A Mallawa C . Development of a treatment protocol for cobra (*Naja naja*) bite envenoming in dogs. Toxins (Basel). (2020) 12:694. doi: 10.3390/toxins12110694, 33147770 PMC7694019

[71] Heller J Bosward KL Hodgson DR Pottie R. Anuric renal failure in a dog after red-bellied black snake (*Pseudechis porphyriacus*) envenomation. Aust Vet J. (2006) 84:158–62. doi: 10.1111/j.1751-0813.2006.tb12769.x, 16739524

[72] Hrovat A Schoeman JP de Laat B Meyer E Smets P Goddard A . Evaluation of snake envenomation-induced renal dysfunction in dogs using early urinary biomarkers of nephrotoxicity. Vet J. (2013) 198:239–44. doi: 10.1016/j.tvjl.2013.06.030, 23916665

[73] Puig J Vilafranca M Font A Closa J Pumarola M Mascort J. Acute intrinsic renal failure and blood coagulation disorders after a snakebite in a dog. J Small Anim Pract. (1995) 36:333–6. doi: 10.1111/j.1748-5827.1995.tb02942.x, 7474966

[74] Harjen HJ Anfinsen KP Hultman J Moldal ER Szlosek D Murphy R . Evaluation of urinary clusterin and cystatin B as biomarkers for renal injury in dogs envenomated by the European adder (*Vipera berus*). Top Companion Anim Med. (2022) 46:100586. doi: 10.1016/j.tcam.2021.100586, 34583053

[75] Budzynski AZ Pandya BV Rubin RN Brizuela BS Soszka T Stewart GJ. Fibrinogenolytic afibrinogenemia after envenomation by western diamondback rattlesnake (*Crotalus atrox*). Blood. (1984) 63:1–14. 6537796

[76] Xu X Liu Q. Binding of anticoagulation factor II from the venom of *Agkistrodon acutus* with activated coagulation factor X. Toxicon. (2001) 39:1359–65. doi: 10.1016/s0041-0101(01)00088-5, 11384724

[77] Zhang Y Xu X Shen D Song J Guo M Yan X. Anticoagulation factor I, a snaclec (snake C-type lectin) from *Agkistrodon acutus* venom binds to FIX as well as FX: Ca2+ induced binding data. Toxicon. (2012) 59:718–23. doi: 10.1016/j.toxicon.2012.03.006 22445822

[78] Zingali RB Jandrot-Perrus M Guillin MC Bon C. Bothrojaracin, a new thrombin inhibitor isolated from *Bothrops jararaca* venom: characterization and mechanism of thrombin inhibition. Biochemistry. (1993) 32:10794–802. doi: 10.1021/bi00091a034, 8399228

[79] Lee WH Du XY Lu QM Clemetson KJ Zhang Y. Stejnulxin, a novel snake C-type lectin-like protein from *Trimeresurus stejnegeri* venom is a potent platelet agonist acting specifically via GPVI. Thromb Haemost. (2003) 90:662–71. 14515187 10.1160/TH03-05-0269

[80] Polgar J Clemetson JM Kehrel BE Wiedemann M Magnenat EM Wells TN . Platelet activation and signal transduction by convulxin, a C-type lectin from *Crotalus durissus terrificus* (tropical rattlesnake) venom via the p62/GPVI collagen receptor. J Biol Chem. (1997) 272:13576–83. 9153205 10.1074/jbc.272.21.13576

[81] Tai H Wei Q Jin Y Su M Song JX Zhou XD . TMVA, a snake C-type lectin-like protein from *Trimeresurus mucrosquamatus* venom, activates platelet via GPIb. Toxicon. (2004) 44:649–56. doi: 10.1016/j.toxicon.2004.07.022, 15501291

[82] Eramanis LM Woodward A Courtman N Hughes D Padula A Winkel KD . Coagulation factor activity patterns of venom-induced consumption coagulopathy in naturally occurring tiger snake (*Notechis scutatus*) envenomed dogs treated with antivenom. Toxicon. (2020) 181:36–44. doi: 10.1016/j.toxicon.2020.03.010, 32330462

[83] Holloway SA Parry BW. Observations on blood coagulation after snakebite in dogs and cats. Aust Vet J. (1989) 66:364–6. doi: 10.1111/j.1751-0813.1989.tb09734.x, 2619650

[84] Hackett TB Wingfield WE Mazzaferro EM Benedetti JS. Clinical findings associated with prairie rattlesnake bites in dogs: 100 cases (1989-1998). J Am Vet Med Assoc. (2002) 220:1675–80. doi: 10.2460/javma.2002.220.1675, 12051509

[85] Dickinson CE Traub-Dargatz JL Dargatz DA Bennett DG Knight AP. Rattlesnake venom poisoning in horses: 32 cases (1973-1993). J Am Vet Med Assoc. (1996) 208:1866–71. 8675476

[86] Machado M Wilson TM de Ribeiro Sousa DE Lopes Camara AC Furlan FH Silva Almeida EMJT . Fatal lancehead pit viper (*Bothrops* spp.) envenomation in horses. Toxicon. (2019) 170:41–50. 31499078 10.1016/j.toxicon.2019.09.002

[87] Fielding CL Pusterla N Magdesian KG Higgins JC Meier CA. Rattlesnake envenomation in horses: 58 cases (1992-2009). J Am Vet Med Assoc. (2011) 238:631–5. doi: 10.2460/javma.238.5.631, 21355806

[88] de Sousa ALV de Sousa DER de Macedo IL Albuquerque Cerqueira L da Fonseca YNG Oliveira AB . Intracerebral hemorrhage (hemorrhagic stroke) secondary to *Bothrops* spp. snakebite envenomation in a horse. Toxicon. (2025) 263:108408. doi: 10.1016/j.toxicon.2025.108408, 40379034

[89] Lawler JB Frye MA Bera MM Ehrhart EJ Bright JM. Third-degree atrioventricular block in a horse secondary to rattlesnake envenomation. J Vet Intern Med. (2008) 22:486–90. doi: 10.1111/j.1939-1676.2008.0067.x, 18371038

[90] Bart G Pineau S Biron C Connault J Artifoni M. Bilateral pulmonary embolism following a viper envenomation in France: a case report and review. Medicine. (2016) 95:e2798. doi: 10.1097/MD.0000000000002798, 27175626 PMC4902468

[91] Bhagat R Sharma K Sarode R Shen Y-M. Delayed massive pulmonary thromboembolic phenomenon following envenomation by Mojave rattlesnake (*Crotalus scutulatus*). Thromb Haemost. (2010) 104:186–8. doi: 10.1160/th09-12-0874, 20390235

[92] Makis A Kattamis A Grammeniatis V Sihlimiri P Kotsonis H Iliadis A . Pulmonary embolism after snake bite in a child with diamond-Blackfan anemia. J Pediatr Hematol Oncol. (2011) 33:68–70. doi: 10.1097/MPH.0b013e3181e88677, 20881870

[93] Rathnayaka R Ranathunga P Kularatne SAM. Pulmonary hemorrhage and the management following Russell’s viper (*Daboia russelii*) envenoming in Sri Lanka. Asia Pacific J Med Toxicol. (2020) 9:72–6.

[94] Vasconez-Gonzalez J Noboa-Lasso ML Ortiz-Prado E. Snake venom and cerebrovascular events: insights and public health implications. Front Public Health. (2025) 13:1513453. doi: 10.3389/fpubh.2025.1513453, 39975792 PMC11836001

[95] Rodriguez C Estrada R Herrera M Gomez A Segura A Vargas M . *Bothrops asper* envenoming in cattle: clinical features and management using equine-derived whole IgG antivenom. Vet J. (2016) 207:160–3. 27152384 10.1016/j.tvjl.2015.08.008

[96] Berrocal A Gutierrez J Estrada R. Snake envenomation in bovine. Large Animal Practice. (1998) 19:26–27. Available online at: https://europepmc.org/abstract/AGR/IND21811739

[97] Shrikant SC Kaka JR Udhavrao BA Suryakant MP Neelam K. Epidemiological, clinical and hematobiochemical studies on hemotoxic snakebite in bovines. Indian J Anim Sci. (2023) 93:23–8. doi: 10.56093/ijans.v93i1.124168

[98] Méndez M Riet-Correa F. Snakebite in sheep. Vet Hum Toxicol. (1995) 37:62–3. 7709596

[99] Leal MdR Aires AR Fillapi A Trost ME. Clinical and pathological observations associated with snake envenomation in two sheep. Acta Sci Vet. (2013) 41:1–4. Available online at: http://www.ufrgs.br/actavet/41-suple-1/CR_32.pdf

[100] Smith J Kovalik D Varga A. Rattlesnake envenomation in three dairy goats. Case Rep Vet Med. (2015) 2015:787534

[101] Garcês A Pereira C Santiago MI Prada J Silva F Pires I. Snakebites in domestic animals. Biol Life Sci Forum. (2023) 24:4.

[102] Agab H. Diseases and causes of mortality in a camel (*Camelus dromedarius*) dairy farm in Saudi Arabia. J Camel Pract Res. (2006) 13:165.

[103] Mirtschin P Masci P Paton D Kuchel T. Snake bites recorded by veterinary practices in Australia. Aust Vet J. (1998) 76:195–8. doi: 10.1111/j.1751-0813.1998.tb10128.x, 9578756

[104] Dykgraaf S Pusterla N Van Hoogmoed LM. Rattlesnake envenomation in 12 New World camelids. J Vet Intern Med. (2006) 20:998–1002. 16955829 10.1892/0891-6640(2006)20[998:reinwc]2.0.co;2

[105] Sonis JM Hackett ES Callan RJ Holt TN Hackett TB. Prairie rattlesnake envenomation in 27 New World camelids. J Vet Intern Med. (2013) 27:1238–41. doi: 10.1111/jvim.12143, 23889704

[106] Cummings CO Eisenbarth JM. Snakebite envenoming in avian species: a systematic scoping review and practitioner experience survey. J Avian Med Surg. (2023) 37:118–31. doi: 10.1647/22-00035, 37733451 PMC10787666

[107] Gilliam LL Brunker J. North American snake envenomation in the dog and cat. Vet Clin North Am Small Anim Pract. (2011) 41:1239–59. doi: 10.1016/j.cvsm.2011.08.008, 22041214

[108] Gutierrez JM Calvete JJ Habib AG Harrison RA Williams DJ Warrell DA. Snakebite envenoming. Nat Rev Dis Primers. (2017) 3:17063 28980622 10.1038/nrdp.2017.79

[109] de Roodt AR Paniagua-Solis JF Dolab JA Estevez-Ramirez J Ramos-Cerrillo B Litwin S . Effectiveness of two common antivenoms for north, central, and south American *Micrurus* envenomations. J Toxicol Clin Toxicol. (2004) 42:171–8. 15214622 10.1081/clt-120030943

[110] Sanchez EE Lopez-Johnston JC Rodriguez-Acosta A Perez JC. Neutralization of two north American coral snake venoms with United States and Mexican antivenoms. Toxicon. (2008) 51:297–303. doi: 10.1016/j.toxicon.2007.10.004, 18054059 PMC3293456

[111] Pizon AFR Ruha AM . Antidotes in depth in Goldfrank’s toxicologic emergencies. 8th ed. New York: McGraw-Hill (2006). 1657 p.

[112] Carotenuto SE Bergman PJ Ray JR McKee T. Retrospective comparison of three antivenoms for the treatment of dogs with crotalid envenomation. J Am Vet Med Assoc. (2021) 259:503–9. doi: 10.2460/javma.259.5.503, 34388014

[113] Otero-Patino R Cardoso JL Higashi HG Nunez V Diaz A Toro MF . A randomized, blinded, comparative trial of one pepsin-digested and two whole IgG antivenoms for *Bothrops* snake bites in Uraba, Colombia. The regional group on Antivenom therapy research (REGATHER). Am J Trop Med Hyg. (1998) 58:183–9. 9580075 10.4269/ajtmh.1998.58.183

[114] Otero-Patino R Segura A Herrera M Angulo Y Leon G Gutierrez JM . Comparative study of the efficacy and safety of two polyvalent, caprylic acid fractionated [IgG and F(ab')2] antivenoms, in *Bothrops asper* bites in Colombia. Toxicon. (2012) 59:344–55. doi: 10.1016/j.toxicon.2011.11.017, 22146491

[115] Squaiella-Baptistao CC Marcelino JR Ribeiro da Cunha LE Gutierrez JM Tambourgi DV. Anticomplementary activity of horse IgG and F(ab')2 antivenoms. Am J Trop Med Hyg. (2014) 90:574–84. doi: 10.4269/ajtmh.13-0591, 24445201 PMC3945706

[116] Eursakun S Simsiriwong P Ratanabanangkoon K. Studies on the fractionation of equine antivenom IgG by combinations of ammonium sulfate and caprylic acid. Toxicon. (2012) 60:1022–9. doi: 10.1016/j.toxicon.2012.07.005, 22842065

[117] Otero R Gutierrez JM Rojas G Nunez V Diaz A Miranda E . A randomized blinded clinical trial of two antivenoms, prepared by caprylic acid or ammonium sulphate fractionation of IgG, in *Bothrops* and *Porthidium* snake bites in Colombia: correlation between safety and biochemical characteristics of antivenoms. Toxicon. (1999) 37:895–908. 10340829 10.1016/s0041-0101(98)00220-7

[118] Boyer LV Seifert SA Clark RF McNally JT Williams SR Nordt SP . Recurrent and persistent coagulopathy following pit viper envenomation. Arch Intern Med. (1999) 159:706–10. doi: 10.1001/archinte.159.7.706, 10218750

[119] Dart RC Seifert SA Carroll L Clark RF Hall E Boyer-Hassen LV . Affinity-purified, mixed monospecific crotalid antivenom ovine fab for the treatment of crotalid venom poisoning. Ann Emerg Med. (1997) 30:33–9. doi: 10.1016/s0196-0644(97)70107-0, 9209222

[120] Harjen HJ Hellum M Rortveit R Oscarson M Anfinsen KP Moldal ER . Persistent hypercoagulability in dogs envenomated by the European adder (*Vipera berus berus*). PLoS One. (2022) 17:e0263238. doi: 10.1371/journal.pone.0263238, 35180240 PMC8856559

[121] Schaer M. Persistent pit viper envenomation in three dogs. Toxicon. (2019) 166:83–7. doi: 10.1016/j.toxicon.2019.05.013, 31129161

[122] Anlén KG. Effects of bites by the European adder (*Vipera berus*) in seven Swedish horses. Vet Rec. (2008) 162:652–6. doi: 10.1136/vr.162.20.652, 18487585

[123] de Acosta Perez O Teibler P Leiva L Rios E Sanchez Negrette M Pollitt C. Equine laminitis: bites by *Bothrops* spp cause hoof lamellar pathology in the contralateral as well as in the bitten limb. Toxicon. (2006) 48:307–12. 16890973 10.1016/j.toxicon.2006.06.010

[124] Migliorisi A Hassel DM Moore AR Blair BW Wilkins PA. Viscoelastic testing is improved following antivenom treatment in rattlesnake-envenomated equids. Am J Vet Res. (2025) 86:1–9. doi: 10.2460/ajvr.25.04.0147, 40690938

[125] Peterson ME. Snake bite: pit vipers. Clin Tech Small Anim Pract. (2006) 21:174–82. doi: 10.1053/j.ctsap.2006.10.008, 17265901

[126] Iliyasu G Dayyab FM Michael GC Hamza M Habib MA Gutierrez JM . Case fatality rate and burden of snakebite envenoming in children - a systematic review and meta-analysis. Toxicon. (2023) 234:107299. doi: 10.1016/j.toxicon.2023.107299, 37739273

[127] Janes DN Jr Bush SP Kolluru GR. Large snake size suggests increased snakebite severity in patients bitten by rattlesnakes in Southern California. Wilderness Environ Med. (2010) 21:120–6. doi: 10.1016/j.wem.2010.01.010, 20591373

[128] Seifert SA Armitage JO Sanchez EE. Snake envenomation. N Engl J Med. (2022) 386:68–78. doi: 10.1056/NEJMra2105228, 34986287 PMC9854269

[129] Southern CJ Allen-Durrance AE Schaer M. Pit viper envenomation in pediatric dogs: 5 cases. Clin Case Rep. (2021) 9:e05065. doi: 10.1002/ccr3.5065, 34804530 PMC8588840

[130] Smith CF Nikolakis ZL Ivey K Perry BW Schield DR Balchan NR . Snakes on a plain: biotic and abiotic factors determine venom compositional variation in a wide-ranging generalist rattlesnake. BMC Biol. (2023) 21:136. doi: 10.1186/s12915-023-01626-x, 37280596 PMC10246093

[131] Pashmakova MB Bishop MA Black DM Bernhard C Johnson SI Mensack S . Multicenter evaluation of the administration of crotalid antivenom in cats: 115 cases (2000-2011). J Am Vet Med Assoc. (2013) 243:520–5. doi: 10.2460/javma.243.4.520, 23902445

[132] Peterson ME Matz M Seibold K Plunkett S Johnson S Fitzgerald K. A randomized multicenter trial of Crotalidae polyvalent immune F(ab) antivenom for the treatment of rattlesnake envenomation in dogs. J Vet Emerg Crit Care (San Antonio). (2011) 21:335–45. doi: 10.1111/j.1476-4431.2011.00643.x 21827591

[133] Lavonas EJ Ruha AM Banner W Bebarta V Bernstein JN Bush SP . Unified treatment algorithm for the management of crotaline snakebite in the United States: results of an evidence-informed consensus workshop. BMC Emerg Med. (2011) 11:2. doi: 10.1186/1471-227x-11-2, 21291549 PMC3042971

[134] Armentano RA Schaer M. Overview and controversies in the medical management of pit viper envenomation in the dog. J Vet Emerg Crit Care (San Antonio). (2011) 21:461–70. doi: 10.1111/j.1476-4431.2011.00677.x, 22316194

[135] Katzenbach JE Foy DS. Retrospective evaluation of the effect of antivenom administration on hospitalization duration and treatment cost for dogs envenomated by *Crotalus viridis*: 113 dogs (2004-2012). J Vet Emerg Crit Care (San Antonio). (2015) 25:655–9. doi: 10.1111/vec.12349, 26260356

[136] McCown JL Cooke KL Hanel RM Jones GL Hill RC. Effect of antivenin dose on outcome from crotalid envenomation: 218 dogs (1988-2006). J Vet Emerg Crit Care (San Antonio). (2009) 19:603–10. doi: 10.1111/j.1476-4431.2009.00487.x, 20017766

[137] Sutton NM Bates N Campbell A. Canine adder bites in the UK: a retrospective study of cases reported to the veterinary poisons information service. Vet Rec. (2011) 169:607. doi: 10.1136/vr.d4695, 21868437

[138] Migliorisi A. Multicenter, prospective evaluation of the clinical efficacy and safety of F(ab’)2 antivenom administered to snake envenomated horses in the United States In: Proceedings of the American Association of equine practitioners. Denver: (2025)

[139] Isbister GK. The critical time period for administering antivenom: golden hours and missed opportunities. Clin Toxicol (Phila). (2024) 62:277–9. doi: 10.1080/15563650.2024.2352026, 38804828

[140] Bishop RC McCoy AM Kemper AM Stewart RM Wilkins PA. Short-term administration of flunixin meglumine or firocoxib does not alter viscoelastic coagulation profiles in healthy horses. J Am Vet Med Assoc. (2022) 260:1963–6. doi: 10.2460/javma.22.08.0367, 36198050

[141] Gutierrez JM Leon G Lomonte B. Pharmacokinetic-pharmacodynamic relationships of immunoglobulin therapy for envenomation. Clin Pharmacokinet. (2003) 42:721–41. 12846594 10.2165/00003088-200342080-00002

[142] Brandeker E Hillstrom A Hanas S Hagman R Holst BS. The effect of a single dose of prednisolone in dogs envenomated by *Vipera berus*--a randomized, double-blind, placebo-controlled clinical trial. BMC Vet Res. (2015) 11:44. doi: 10.1186/s12917-015-0352-6, 25886633 PMC4349773

[143] Dorooshi G Javid ZN Meamar R Farjzadegan Z Nasri M Eizadi-Mood N. Evaluation of the effects of anti-inflammatory drugs on local and systemic manifestations of snakebite: a cross-sectional study. J Venom Res. (2021) 11:21–5. 34123361 PMC8169030

[144] Ghosh M Acharyya A Bhattacharya P Chakrabortty S. Role of steroid on management of limb swelling and local pain in haematotoxic snake bite. J Family Med Prim Care. (2022) 11:7394–7. 36993125 10.4103/jfmpc.jfmpc_1371_22 PMC10041249

[145] Lenchner I Aroch I Segev G Kelmer E Bruchim Y. A retrospective evaluation of *Vipera palaestinae* envenomation in 18 cats: (2006-2011). J Vet Emerg Crit Care (San Antonio). (2014) 24:437–43. doi: 10.1111/vec.12207, 25154358

[146] Witsil AJ Wells RJ Woods C Rao S. 272 cases of rattlesnake envenomation in dogs: demographics and treatment including safety of F(ab')2 antivenom use in 236 patients. Toxicon. (2015) 105:19–26. doi: 10.1016/j.toxicon.2015.08.028, 26341419

[147] Szold O Ben-Abraham R Frolkis I Sorkine M Sorkine P. Tumor necrosis factor as a mediator of cardiac toxicity following snake envenomation. Crit Care Med. (2003) 31:1449–53. doi: 10.1097/01.ccm.0000050440.87890.92, 12771617

[148] Szold O Ben-Abraham R Weinbroum AA Englender TE Ovadia D Sorkine M . Antagonization of TNF attenuates systemic hemodynamic manifestations of envenomation in a rat model of *Vipera aspis* snakebite. Intensive Care Med. (2001) 27:884–8. doi: 10.1007/s001340100875, 11430545

[149] Bonilla CA Rammel OJ. Comparative biochemistry and pharmacology of salivary gland secretions. III. Chromatographic isolation of a myocardial depressor protein (MDP) from the venom of *Crotalus atrox*. J Chromatogr. (1976) 124:303–14. 965463 10.1016/s0021-9673(00)89745-9

[150] Holstege CP Miller MB Wermuth M Furbee B Curry SC. Crotalid snake envenomation. Crit Care Clin. (1997) 13:889–921. doi: 10.1016/s0749-0704(05)70373-0, 9330845

[151] White J. Snake venoms and coagulopathy. Toxicon. (2005) 45:951–67. doi: 10.1016/j.toxicon.2005.02.030, 15922768

[152] Ledbetter EO Kutscher AE. The aerobic and anaerobic flora of rattlesnake fangs and venom: therapeutic implications. Arch Environ Health. (1969) 19:770–8. 4900686 10.1080/00039896.1969.10666929

[153] August JA Boesen KJ Hurst NB Shirazi FM Klotz SA. Prophylactic antibiotics are not needed following rattlesnake bites. Am J Med. (2018) 131:1367–71. doi: 10.1016/j.amjmed.2018.06.006, 30392637

[154] Carr A Schultz J. Prospective evaluation of the incidence of wound infection in rattlesnake envenomation in dogs. J Vet Emerg Crit Care (San Antonio). (2015) 25:546–51. doi: 10.1111/vec.12337, 26112434

[155] Perumal Samy R Gopalakrishnakone P Ho B Chow VT. Purification, characterization and bactericidal activities of basic phospholipase A2 from the venom of Agkistrodon halys (Chinese pallas). Biochimie. (2008) 90:1372–88. doi: 10.1016/j.biochi.2008.04.007, 18472013

[156] Stiles BG Sexton FW Weinstein SA. Antibacterial effects of different snake venoms: purification and characterization of antibacterial proteins from *Pseudechis australis* (Australian king brown or Mulga snake) venom. Toxicon. (1991) 29:1129–41. doi: 10.1016/0041-0101(91)90210-i, 1796476

[157] Talan DA Citron DM Overturf GD Singer B Froman P Goldstein EJ. Antibacterial activity of crotalid venoms against oral snake flora and other clinical bacteria. J Infect Dis. (1991) 164:195–8. doi: 10.1093/infdis/164.1.195, 2056205

[158] Brenes-Chacon H Ulloa-Gutierrez R Soriano-Fallas A Camacho-Badilla K Valverde-Munoz K Avila-Aguero ML. Bacterial infections associated with Viperidae snakebites in children: a 14-year experience at the hospital Nacional de Ninos de Costa Rica(dagger). Am J Trop Med Hyg. (2019) 100:1227–9. 30915952 10.4269/ajtmh.18-1015 PMC6493952

[159] Houcke S Resiere D Lontsingoula GR Cook F Lafouasse P Pujo JM . Characteristics of snakebite-related infection in French Guiana. Toxins (Basel). (2022) 14. doi: 10.3390/toxins14020089, 35202117 PMC8878173

[160] Wood D Sartorius B Hift R. Ultrasound findings in 42 patients with cytotoxic tissue damage following bites by South African snakes. Emerg Med J. (2016) 33:477–81. doi: 10.1136/emermed-2015-205279, 27068867

[161] Morris CAD Donaldson RE. Mechanical ventilation in snake envenomation of dogs and cats. Front Vet Sci. (2023) 10:1071257. doi: 10.3389/fvets.2023.1071257, 37065246 PMC10090310

[162] Ong HM Kelers K Hughes D Boller M. Retrospective evaluation of cats with elapid snake envenomation associated neurotoxicity requiring mechanical ventilation: 12 cases (2005–2014). J Vet Emerg Crit Care. (2017) 27:579–85. doi: 10.1111/vec.12632, 28799698

[163] Trigg N Leister E Whitney J McAlees T. Outcomes of mechanical ventilation in 302 dogs and cats in Australia (2005-2013). Aust Vet Pract. (2014) 44:698–703.

[164] Lee JM Song JH Song KH. A retrospective evaluation of snake envenomation in dogs in South Korea (2004-2021). Toxins (Basel). (2022) 14:565. doi: 10.3390/toxins14080565, 36006225 PMC9415592

[165] Nascimento TP Vilhena Silva-Neto A Baia-da-Silva DC da Silva Balieiro PC Baleiro A Sachett J . Pregnancy outcomes after snakebite envenomations: a retrospective cohort in the Brazilian Amazonia. PLoS Negl Trop Dis. (2022) 16:e0010963 36469516 10.1371/journal.pntd.0010963 PMC9754599

[166] Langley RL. Snakebite during pregnancy: a literature review. Wilderness Environ Med. (2010) 21:54–60. doi: 10.1016/j.wem.2009.12.025, 20591355

[167] Gabriel-Robez O Clavert J. Teratogenic and lethal properties of the various fractions of venom of the viper, *Vipera aspis*. Cells Tissues Organs. (1980) 108:226–9. doi: 10.1159/000145303, 7405539

[168] Mohamed A Nawar N Hanna M. Some effects of *Naja nigricollis* envenomation on developing fetal tissue. Toxicon. (1974) 12:477–8. doi: 10.1016/0041-0101(74)90036-1, 4460283

[169] Altuğ N İşler CT. Snake envenomation in two cattle: clinical/laboratory aspects and treatment using equine-derived antivenin of Viperidae. Turk J Vet Anim Sci. (2019) 43:546–50.

[170] Hedges K Schaer M Allen-Durrance A. Pit viper envenomation in two pregnant bitches. J Am Anim Hosp Assoc. (2024) 60:114–9. doi: 10.5326/jaaha-ms-7377, 38662995

[171] Hawgood BJ. Doctor Albert Calmette 1863-1933: founder of antivenomous serotherapy and of antituberculous BCG vaccination. Toxicon. (1999) 37:1241–58. doi: 10.1016/s0041-0101(99)00086-0 10400286

[172] Sewall H. Experiments on the preventive inoculation of rattlesnake venom. J Physiol. (1887) 8:203–10. doi: 10.1113/jphysiol.1887.sp000253, 16991478 PMC1485134

[173] Gutierrez JM. Global availability of antivenoms: the relevance of public manufacturing laboratories. Toxins (Basel). (2018) 11:5. doi: 10.3390/toxins11010005, 30586868 PMC6356591

[174] Choraria A Somasundaram R Gautam M Ramanathan M Paray BA Al-Sadoon MK . Experimental antivenoms from chickens and rabbits and their comparison with commercially available equine antivenom against the venoms of Daboia russelii and *Echis carinatus* snakes. Toxin Rev. (2020) 40:702–13. doi: 10.1080/15569543.2020.1756858

[175] Choraria A Somasundaram R Janani S Rajendran S Oukkache N Michael A. Chicken egg yolk antibodies (IgY)-based antivenom for neutralization of snake venoms: a review. Toxin Rev. (2021) 41:1018–29. doi: 10.1080/15569543.2021.1942063

[176] Duan HL He QY Zhou B Wang WW Li B Zhang YZ . Anti-*Trimeresurus albolabris* venom IgY antibodies: preparation, purification and neutralization efficacy. J Venom Anim Toxins Incl Trop Dis. (2016) 22:23. doi: 10.1186/s40409-016-0078-3, 27563307 PMC4997716

[177] Kpordze SW Mobegi VA Kikuvi GM Gikunju JK Setsoafia Saba CK Moshe J . Generation of chicken-based IgY polyclonal antibodies against Dendroaspis polylepis and preclinical evaluation of envenomation-neutralizing efficacy vis-a-vis selected commercial antivenoms. Toxicon X. (2024) 23:100201. doi: 10.1016/j.toxcx.2024.100201, 39050508 PMC11267070

[178] Liu J He Q Wang W Zhou B Li B Zhang Y . Preparation and neutralization efficacy of IgY antibodies raised against *Deinagkistrodon acutus* venom. J Venom Anim Toxins Incl Trop Dis. (2017) 23:22. doi: 10.1186/s40409-017-0112-0, 28396683 PMC5379703

[179] Adriao AAX Dos Santos AO de Lima E Maciel JB Paz WHP da Silva FMA . Plant-derived toxin inhibitors as potential candidates to complement antivenom treatment in snakebite envenomations. Front Immunol. (2022) 13:842576. doi: 10.3389/fimmu.2022.842576, 35615352 PMC9126284

[180] Gomes M Alvarez MA Quellis LR Becher ML Castro JMA Gameiro J . Expression of an scFv antibody fragment in Nicotiana benthamiana and in vitro assessment of its neutralizing potential against the snake venom metalloproteinase BaP1 from *Bothrops asper*. Toxicon. (2019) 160:38–46. 30802471 10.1016/j.toxicon.2019.02.011

[181] Julve Parreno JM Huet E Fernandez-Del-Carmen A Segura A Venturi M Gandia A . A synthetic biology approach for consistent production of plant-made recombinant polyclonal antibodies against snake venom toxins. Plant Biotechnol J. (2018) 16:727–36. doi: 10.1111/pbi.12823, 28850773 PMC5814581

[182] Greenberg AS Avila D Hughes M Hughes A McKinney EC Flajnik MF. A new antigen receptor gene family that undergoes rearrangement and extensive somatic diversification in sharks. Nature. (1995) 374:168–73. doi: 10.1038/374168a0, 7877689

[183] Hamers-Casterman C Atarhouch T Muyldermans S Robinson G Hamers C Songa EB . Naturally occurring antibodies devoid of light chains. Nature. (1993) 363:446–8. doi: 10.1038/363446a0, 8502296

[184] Revets H De Baetselier P Muyldermans S. Nanobodies as novel agents for cancer therapy. Expert Opin Biol Ther. (2005) 5:111–24. doi: 10.1517/14712598.5.1.111, 15709914

[185] Alirahimi E Kazemi-Lomedasht F Shahbazzadeh D Habibi-Anbouhi M Hosseininejad Chafi M Sotoudeh N . Nanobodies as novel therapeutic agents in envenomation. Biochim Biophys Acta Gen Subj. (2018) 1862:2955–65. doi: 10.1016/j.bbagen.2018.08.019, 30309831

[186] Bailon Calderon H Yaniro Coronel VO Caceres Rey OA Colque Alave EG Leiva Duran WJ Padilla Rojas C . Development of nanobodies against hemorrhagic and myotoxic components of *Bothrops atrox* snake venom. Front Immunol. (2020) 11:655. doi: 10.3389/fimmu.2020.00655, 32457735 PMC7224310

[187] Benard-Valle M Wouters Y Ljungars A Nguyen GTT Ahmadi S Ebersole TW . In vivo neutralization of coral snake venoms with an oligoclonal nanobody mixture in a murine challenge model. Nat Commun. (2024) 15:4310. doi: 10.1038/s41467-024-48539-z, 38773068 PMC11109316

[188] Oghalaie A Hosseininejad-Chafi M Mejri H Zareinejad MR Bouhaouala-Zahar B Bagheri KP . Development and characterization of nanobody against envenomation by *Naja naja oxiana*. Toxicon. (2024) 249:108057. doi: 10.1016/j.toxicon.2024.108057, 39103096

[189] Torres SV Valle MB Mackessy SP Menzies SK Casewell NR Ahmadi S . De novo designed proteins neutralize lethal snake venom toxins. Nature. (2025) 639:225–31. 39814879 10.1038/s41586-024-08393-x PMC11882462

[190] Albulescu LO Xie C Ainsworth S Alsolaiss J Crittenden E Dawson CA . A therapeutic combination of two small molecule toxin inhibitors provides broad preclinical efficacy against viper snakebite. Nat Commun. (2020) 11:6094. doi: 10.1038/s41467-020-19981-6, 33323937 PMC7738508

[191] Gilliam LL Gilliam J Samuel SP Carter RW Ritchey J Bulfone T . Oral and IV varespladib rescue experiments in juvenile pigs with weakness induced by Australian and Papuan *Oxyuranus scutellatus* venoms. Toxins (Basel). (2023) 15:557. doi: 10.3390/toxins15090557, 37755983 PMC10537020

[192] Gerardo CJ Carter RW Kumar S Shirazi FM Kotehal SD Akpunonu PD . Oral varespladib for the treatment of snakebite envenoming in India and the USA (BRAVO): a phase II randomised clinical trial. BMJ Glob Health. (2024) 9:e015985. doi: 10.1136/bmjgh-2024-015985 PMC11499837 39442939

[193] Woliver C Kastenholz V Southern C Odunayo A Schaer M Allen-Durrance A. Phospholipase A2 inhibitor may shorten the duration of clinical signs in the treatment of neurotoxicity caused by eastern coral snake (*Micrurus fulvius*) envenomation in 3 dogs. J Am Vet Med Assoc. (2025) 263:1–7. doi: 10.2460/javma.25.04.0275 40738154

[194] Johnson TE Wells RJ Bell A Nielsen VG Olver CS. Carbon monoxide releasing molecule enhances coagulation and decreases fibrinolysis in canine plasma exposed to *Crotalus viridis* venom in vitro and in vivo. Basic Clin Pharmacol Toxicol. (2019) 125:328–36. doi: 10.1111/bcpt.13242, 31059181

[195] Bava D Kumar PHA Gupta A Mandal S Bajpayee A Gopalakrishnan M . Redefining the role of therapeutic plasma exchange in complications of *Echis carinatus sochureki* envenomation refractory to anti-snake venom: a case series. Asian J Transfus Sci. (2023) 17:295–300. doi: 10.4103/ajts.ajts_49_22, 38274951 PMC10807517

[196] Berber I Korkmaz S Sarici A Erkurt MA Kuku I Kaya E . Therapeutic plasma exchange for envenomation: is it reasonable? Transfus Apher Sci. (2021) 60:103241. doi: 10.1016/j.transci.2021.103241, 34429240

[197] Fouad MM Zawilla NH Abdelsamie AA Manawil M Shehata RSA Mohammed RS . Successful management of severe unresponsive snake bite envenomation using plasmapheresis and corticosteroid at Egyptian national environmental and clinical toxicology research center: a case report. Wilderness Environ Med. (2024) 35:82–7. doi: 10.1177/10806032231225102, 38379491

[198] Mohan G Guduri PR Shastry S. Role of therapeutic plasma exchange in snake bite associated thrombotic microangiopathy-a case report with review of literature. J Clin Apher. (2019) 34:507–9. doi: 10.1002/jca.21691, 30779435

[199] Oliveira ME Campanholi J Cavalcante RL Moreno FS Yoshida EH Dini MMJ . Experimental model for removal of snake venom via hemoperfusion in rats. J Vet Emerg Crit Care (San Antonio). (2020) 30:286–94. doi: 10.1111/vec.12949, 32112523

[200] Savas AM Ozluer YE Buyuktas OC Akkus F. Successful use of cytokine hemadsorption filter in *Montivipera xanthina* envenomation: a case report. Cureus. (2025) 17:e77807. doi: 10.7759/cureus.77807, 39991365 PMC11843179

